# Potential Anti-Inflammatory Constituents from *Aesculus wilsonii* Seeds

**DOI:** 10.3390/molecules29051136

**Published:** 2024-03-03

**Authors:** Ping Zhang, Lequan Yu, Huina Cao, Jingya Ruan, Fei Li, Lijie Wu, Yi Zhang, Tao Wang

**Affiliations:** 1 Tianjin Key Laboratory of TCM Chemistry and Analysis, Tianjin University of Traditional Chinese Medicine, 10 Poyanghu Road, West Area, Tuanbo New Town, Jinghai District, Tianjin 301617, China; zp10259611@163.com (P.Z.); 18822575726@163.com (L.Y.); sxcaonanayy@163.com (H.C.); lf18641423285@163.com (F.L.); wulj0816@163.com (L.W.); 2 Institute of TCM, Tianjin University of Traditional Chinese Medicine, 10 Poyanghu Road, West Area, Tuanbo New Town, Jinghai District, Tianjin 301617, China; ruanjingya@tjutcm.edu.cn

**Keywords:** *Aesculus wilsonii* Rehd, chemical constituents, anti-inflammation, NO release inhibitory effect, TNF-α, IL-1β

## Abstract

A chemical study of *Aesculus wilsonii* Rehd. (also called Suo Luo Zi) and the in vitro anti-inflammatory effects of the obtained compounds was conducted. Retrieving results through SciFinder showed that there were four unreported compounds, aeswilosides I–IV (**1**–**4**), along with fourteen known isolates (**5**–**18**). Their structures were elucidated by extensive spectroscopic methods such as UV, IR, NMR, [α]_D_, and MS spectra, as well as acid hydrolysis. Among the known ones, compounds **5**, **6**, **8**–**10**, and **12**–**16** were obtained from the *Aesculus* genus for the first time; compounds **7**, **11**, **17**, and **18** were first identified from this plant. The NMR data of **5** and **18** were reported first. The effects of **1**–**18** on the release of nitric oxide (NO) from lipopolysaccharide (LPS)-induced RAW264.7 cells were determined. The results showed that at concentrations of 10, 25, and 50 μM, the novel compounds, aeswilosides I (**1**) and IV (**4**), along with the known ones, 1-(2-methylbutyryl)phloroglucinyl-glucopyranoside (**10**) and pisuminic acid (**15**), displayed significant inhibitory effects on NO production in a concentration-dependent manner. It is worth mentioning that compound **10** showed the best NO inhibitory effect with a relative NO production of 88.1%, which was close to that of the positive drug dexamethasone. The Elisa experiment suggested that compounds **1**, **4**, **10**, and **15** suppressed the release of TNF-α and IL-1β as well. In conclusion, this study enriches the spectra of compounds with potential anti-inflammatory effects in *A. wilsonii* and provides new references for the discovery of anti-inflammatory lead compounds, but further mechanistic research is still needed.

## 1. Introduction

Inflammation is often associated with the development and progression of cancer, immune system disorder, Alzheimer disease, depression, etc. [1]. Therefore, easing inflammation plays a crucial role in the prevention and treatment of a variety of diseases. Current anti-inflammatory drugs primarily consist of steroidal and non-steroidal agents [2]. Clinical studies have demonstrated that prolonged use of steroidal anti-inflammatory drugs can result in osteoporosis in 50% of patients [3]. Non-steroidal anti-inflammatory drugs also exhibit notable adverse reactions, including peptic ulcers, renal failure, and cardiovascular diseases [4]. Traditional Chinese medicine (TCM) has garnered significant attention from researchers due to its remarkable efficacy and minimal side effects.

*Aesculus wilsonii* Rehd. (*Aesculus* genus, Hippocastanaceae family) is mainly used in TCM to cure liver and stomach *qi* stagnation, chest and abdominal distension, and stomach pain, and has been found to show various activities such as anti-inflammatory [5], anti-edema [6], anti-viral [7], and anti-tumor [8]. In our previous studies, its nitrogenous compounds [9], saponins [10], and flavonoids [11] were found to exert anti-inflammatory activity through the suppression of MAPK (P38), nuclear factor kappa-B (NF-κB), and the signal transducer and activator of transcription 3 (STAT3) cross-talk signaling pathways or the NF-κB and NLR family pyrin domain containing 3 (NLRP3) pathway. These findings suggest that *A. wilsonii* seeds are a good source for discovering anti-inflammatory constituents. This study focuses on the rarely reported potential of active phenolic acids and organic acids and is a supplement to previous research.

RAW 264.7 cells are a type of mouse macrophage commonly used for studying in vitro inflammation, immunity, etc. [12]. Lipopolysaccharides (LPS) can induce acute inflammatory responses, releasing inflammatory factors such as nitric oxide (NO) and interleukin [13]. In recent years, the macrophage inflammation model, established by inducing RAW264.7 cells with LPS, has been widely used for the preliminary screening of anti-inflammatory activities and their pathways of action [14], with simple operation and strong universality.

**Figure 1:**
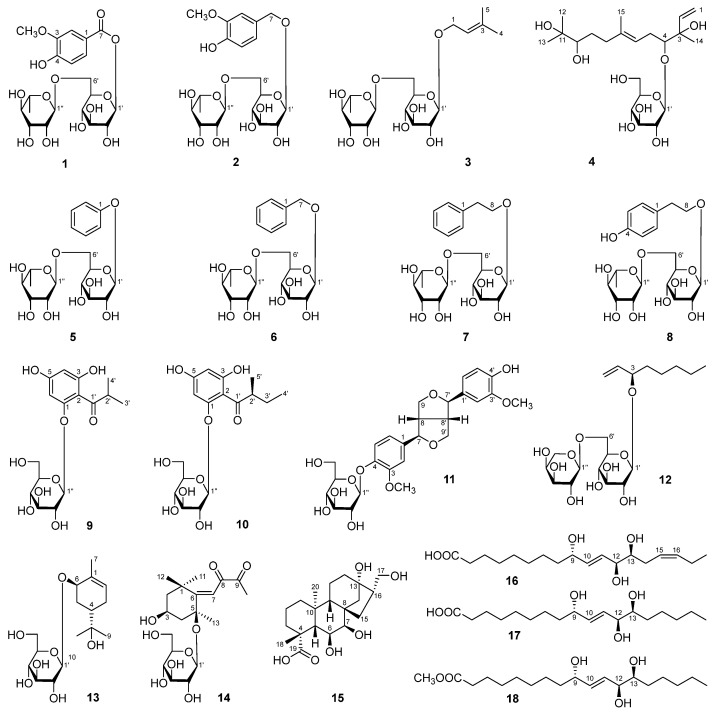
The structures of compounds **1**–**18** obtained from *A. wilsonii* seeds.

In the ongoing search for these kinds of bioactive components, many chromatographic materials, such as D101 resin, silica gel, ODS, MCI gel CHP 20P, and Sephadex LH-20 column chromatographies (CC), as well as preparative high-performance liquid chromatography (pHPLC), were used to fractionate and purify the compounds from the 70% ethanol extraction of *A. wilsonii* seeds. Various spectroscopic techniques, including UV, IR, NMR, [α]_D_, and MS spectra, were applied to identify the structure of the isolates. As results, eighteen compounds were identified from it. Retrieving results using SciFinder showed that there were four unreported compounds, named as aeswilosides I–IV (**1**–**4**), and fourteen known ones, including phenyl-*O*-α-l-rhamnopyranosyl(1→6)-β-d-glucopyranoside (**5**), benzyl-*O*-α-l-rhamnopyranosyl(1→6)-β-d-glucopyranoside (**6**) [15], 2-phenethyl-*O*-α-l-rhamnopyranosyl(1→6)-β-d-glucopyranoside (**7**) [15], asechipuroside A (**8**) [16], 1-[(2-methylpropanoyl)phloroglucinyl]-β-d-glucopyranoside (**9**) [17], 1-(2-methylbutyryl)phloroglucinyl-glucopyranoside (**10**) [18], (–)-pinoresinol 4-*O*-β-d-glucoside (**11**) [19,20], 3-*O*-[α-l-arabinopyranosyl(1→6)-β-d-glucopyranosyl]oct-1-ene-3-ol (**12**) [21], myrseguinoside A (**13**) [22], lippianoside E (**14**) [23], pisuminic acid (**15**) [24], fulgidic acid (**16**) [25], (9*S*,10*E*,12*S*,13*S*)-9,12,13-trihydroxy-10-octadecenoic acid (**17**) [26], and methyl (9*S*,10*E*,12*S*,13*S*)-9,12,13-trihydroxy-10-octadecenoate (**18**) (Figure 1).

Additionally, an LPS-induced RAW264.7 macrophage model was established to assess the anti-inflammatory efficacy of the identified compounds through a quantification of NO and interleukin (IL)-1β levels in RAW264.7 cells.

## 2. Results

**Figure 2:**
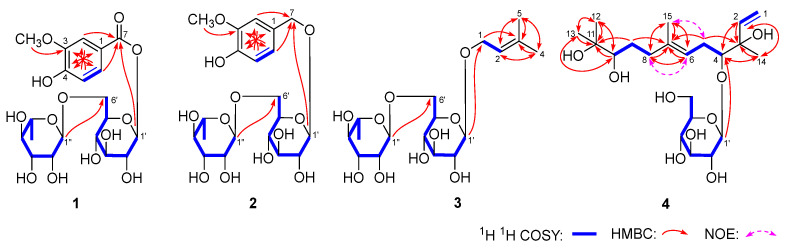
The main ^1^H ^1^H COSY and HMBC correlations of compounds **1**–**4**.

Aeswiloside I (**1**) was obtained as a white powder. The molecular formula of it was assigned as C_20_H_28_O_13_ from the HRESIMS at *m*/*z* 475.14618 [M − H]^−^ (calcd. for C_20_H_27_O_13_, 475.14461). Its IR spectrum presented the characteristic absorptions for hydroxyl (3362 cm^−1^), unsaturated ester carbonyl (1703 cm^−1^), aromatic ring (1599, 1519, 1457 cm^−1^), and ether bond (1067 cm^−1^). The compound underwent hydrolysis using 1 M HCl, and the resulting hydrolysate was subjected to HPLC analysis with a polarimetric detector. As a result, l-rhamnose and d-glucose were identified in it [11]. Combing with the anomeric proton signals at δ_H_ 4.71 (1H, d, *J* = 1.2 Hz, H-1″) and 5.65 (1H, d, *J* = 8.4 Hz, H-1′), as well as the cross peaks shown in the ^1^H ^1^H COSY (Figure 2), the existence of α-l-rhamnopyranosyl and β-d-glucopyranosyl was clarified. In addition, the signals for methoxyl at δ_H_ 3.91 (3H, s, 3-OCH_3_), three aromatic protons with ABX spin coupling systems at δ_H_ 6.86 (1H, d, *J* = 8.4 Hz, H-5), 7.62 (1H, d, *J* = 1.8 Hz, H-2), and 7.65 (1H, dd, *J* = 1.8, 8.4 Hz, H-6)] were detected by its ^1^H NMR spectrum. And the ^13^C NMR (Table 1) spectrum presented one unsaturated ester carbonyl at δ_C_ 166.7 (C-7).

According to the HMBC correlations from δ_H_ 7.62 (H-2), 7.65 (H-6) to δ_C_ 166.7 (C-7); δ_H_ 3.91 (3-OCH_3_) to δ_C_ 148.8 (C-3); δ_H_ 7.62 (H-2) to δ_C_ 125.8 (C-6), 153.4 (C-4); δ_H_ 6.86 (H-5) to δ_C_ 121.8 (C-1), 148.8 (C-3); and δ_H_ 7.65 (H-6) to δ_C_ 114.0 (C-2) 153.4 (C-4) (Figure 2), the aglycon of aeswiloside I (**1**) was elucidated to be 3-methoxy-4-hydroxybenzoic acid. Finally, the linkage location between glycosyl and aglycon, as well as between glycosyls, were determined by the long-range correlations from δ_H_ 5.65 (H-1′) to δ_C_ 166.7 (C-7) and δ_H_ 4.71 (H-1″) to δ_C_ 67.8 (C-6′) (Figure 2).

Aeswiloside II (**2**) was obtained as a white powder with the molecular formula, C_20_H_30_O_12_, gained from the HR-ESI-MS at *m*/*z* 461.16696 [M − H]^−^ (calcd. 461.16535 for C_20_H_29_O_12_). The ^1^H and ^13^C NMR (Table 2) spectra indicated that it had the same groups including one methoxyl, one benzene ring with an ABX spin coupling system, one α-l-rhamnopyranosyl, and one β-d-glucopyranosyl as compound **1**. Compared with **1**, the signal for ester carbonyl disappeared, while the signals at δ_H_ 4.54, 4.77 (1H each, both d, *J* = 11.5 Hz, H_2_-7] for methtylene appeared. Then, the structure of aeswiloside II (**2**) was elucidated according to the HMBC correlations from δ_H_ 6.82 (H-6), 7.03 (H-2) to δ_C_ 71.2 (C-7); δ_H_ 4.29 (H-1′) to δ_C_ 71.2 (C-7); and δ_H_ 4.79 (H-1″) to δ_C_ 68.2 (C-6′) (Figure 2).

Aeswiloside III (**3**) was a white powder. HR-ESI-MS analysis proved its molecular formula to be C_17_H_30_O_10_ through the presence of a peak at *m*/*z* 439.18240 [M + COOH]^−^ (calcd. 439.18100 for C_18_H_31_O_12_). Using a similar method to compound **1**, l-rhamnose and d-glucose were detected from its acid hydrolysate [11]. Compared with aeswilosides I (**1**) and II (**2**), the ^1^H and ^13^C NMR (Table 3) spectra suggested the existence of α-l-rhamnopyranosyl(1→6)-β-d-glucopyranosyl. Moreover, ^1^H, ^13^C NMR and HSQC spectra indicated the existences of two methyls [δ_H_ 1.76, 1.70 (3H each, both s, H_3_-4, 5)], one oxygenated methylene [δ_H_ 4.21, 4.25 (1H each, both dd, *J* = 7.8, 11.4 Hz, H_2_-1)], and one trisubstituted olefin group [δ_H_ 5.36 (1H, m, H-2)].

In its HMBC spectrum, the long-range correlations from δ_H_ 4.21, 4.25 (H_2_-1) to δ_C_ 139.0 (C-3); δ_H_ 5.36 (H-2) to δ_C_ 18.2 (C-5), 26.0 (C-4); δ_H_ 1.76 (H_3_-4) to δ_C_ 18.2 (C-5), 121.5 (C-2), 139.0 (C-3); and δ_H_ 1.70 (H_3_-5) to δ_C_ 26.0 (C-4), 121.5 (C-2), 139.0 (C-3) were displayed (Figure 2), which indicated that its aglycon was 3-methyl-2-butene-1-ol. Finally, the linkage positions between the above-mentioned moieties were elucidated according to the correlations from δ_H_ 4.26 (H-1′) to δ_C_ 66.1 (C-1) and δ_H_ 4.75 (H-1″) to δ_C_ 68.1 (C-6′).

Aeswiloside IV (**4**) was a white powder with a negative optical rotation ([α]_D_^25^ −14.1, MeOH). The molecular formula, C_21_H_38_O_9_ (*m*/*z* 479.25037 [M + COOH]^−^; calcd. for C_22_H_39_O_11_, 479.24869) of it was established on negative-ion HRESIMS analysis. After hydrolyzing compound **4** with 1 M HCl, the product was analyzed by HPLC which was combining with an optical rotation detector [11]. As a result, d-glucose was detected [11]. Its ^1^H NMR (CD_3_OD, 500 MHz) (Table 4) spectrum suggested the presence of one β-d-glucopyranosyl signal at δ_H_ 4.45 (1H, d, *J* = 8.0 Hz, H-1′)], four methyl signals at δ_H_ 1.12, 1.16, 1.27, and 1.60 (3H each, all s, H_3_-12, 13, 14, and 15)], three methylene signals at δ_H_ [2.16 (1H, ddd, *J* = 7.5, 7.5, 15.0 Hz), 2.35 (1H, ddd, *J* = 3.5, 7.5, 15.0 Hz), H_2_-5], [2.05 (1H, ddd, *J* = 8.5, 8.5, 14.0 Hz), 2.24 (1H, ddd, *J* = 4.0, 8.5, 14.0 Hz), H_2_-8], and 1.36, 1.75 (1H each, both m, H_2_-9), two oxygenated methine signals at δ_H_ 3.53 (1H, dd, *J* = 3.5, 9.0, H-4) and 3.25 (1H, m, overlapped, H-10)], one trisubstituted olefinic proton [δ_H_ 5.53 (1H, dd, *J* = 7.5, 7.5 Hz, H-6)], and one pair of terminal olefinic protons [δ_H_ 5.11 (1H, br. d, ca. *J* = 11 Hz), 5.26 (1H, br. d, ca. *J* = 17 Hz), H_2_-1]. Then, the four moieties shown in bold lines in Figure 2 were determined by the cross peaks observed in its ^1^H ^1^H COSY spectrum. The planar structure of aeswiloside IV (**4**) was clarified by the HMBC correlations from δ_H_ 6.05 (H-2) to δ_C_ 24.5 (C-14), 77.2 (C-3), 89.9 (C-4); δ_H_ 3.53 (H-4) to δ_C_ 24.5 (C-14), 142.8 (C-2); δ_H_ 2.16, 2.35 (H_2_-5) to δ_C_ 77.2 (C-3), 136.3 (C-7); δ_H_ 5.53 (H-6) to δ_C_ 16.5 (C-15), 37.9 (C-8), 136.3 (C-7); δ_H_ 2.05, 2.24 (H_2_-8) to δ_C_ 16.5 (C-15), 123.7 (C-6), 136.3 (C-7); δ_H_ 1.36, 1.75 (H_2_-9) to δ_C_ 73.8 (C-11), 136.3 (C-7); δ_H_ 3.25 (H-10) to δ_C_ 24.9 (C-12), 25.9 (C-13), 73.8 (C-11); δ_H_ 1.12 (H_3_-12) to δ_C_ 25.9 (C-13), 73.8 (C-11), 78.9 (C-10); δ_H_ 1.16 (H_3_-13) to δ_C_ 24.9 (C-12), 73.8 (C-11), 78.9 (C-10); δ_H_ 1.27 (H_3_-14) to δ_C_ 77.2 (C-3), 89.9 (C-4), 142.8 (C-2); δ_H_ 1.60 (H_3_-15) to δ_C_ 37.9 (C-8), 123.7 (C-6), 136.3 (C-7); and δ_H_ 4.45 (H-1′) to δ_C_ 89.9 (C-4) (Figure 2), which was a sesquiterpene glycoside. Finally, *Δ*6 was elucidated to be an *E* configuration according to the NOE correlations between δ_H_ 2.16, 2.35 (H_2_-5) and δ_H_ 1.60 (H_3_-15) and δ_H_ 5.53 (H-6) and δ_H_ 2.05, 2.24 (H_2_-8) (Figure 2) as shown in its NOESY spectrum.

The structures of the known compounds **6**–**17** were identified by comparing the spectroscopic data with those reported in the literature. According to the 1D and 2D NMR spectra identification, the NMR data of phenyl-*O*-α-l-rhamnopyranosyl(1→6)-β-d-glucopyranoside (**5**) were firstly assigned. Meanwhile, comparing the ^1^H, ^13^C NMR spectra and the [α]_D_^25^ data with those of compound **17**, the structure of **18** was elucidated to be methyl (9*S*,10*E*,12*S*,13*S*)-9,12,13-trihydroxy-10-octadecenoate; the NMR data of **18** are also first reported here.

The RAW264.7 cells, known for their robust adhesion and phagocytic capacity towards antigens, are widely employed as an in vitro research model for screening bioactive compounds with anti-inflammatory activity. After stimulation by inducers, such as LPS, it will release or cause dissonance of inflammatory factors such as NO, IL-1β, TNF-α, etc. The expression levels of these factors can serve as indicators for in vitro anti-inflammatory activity screening, reflecting the severity of inflammation and commonly used in this context.

**Figure 3:**
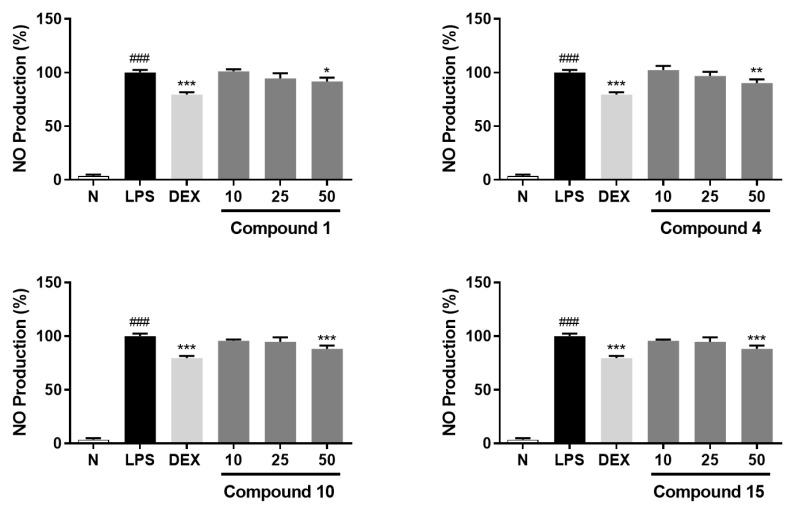
Inhibitory effects of compounds **1**, **4**, **10**, and **15** at concentrations of 10, 25, and 50 μM on NO production in RAW264.7 cells, respectively. Nitrite relative concentration (NRC): percentage of control group (set as 100%). Values represent the mean ± SD of six determinations. * *p* < 0.05; ** *p* < 0.01; *** *p* < 0.001 (Differences between compound-treated group and control group). ^###^ *p* < 0.001 (differences between control group and normal group).

The inhibitory effects of compounds **1**–**18** on NO release were investigated by using LPS-stimulated RAW264.7 cells as an in vitro screening model. Based on the results of the MTT assay (Figure S32), the bioactivity of **1**–**18** at the concentration of 50 μM on the release of NO from RAW264.7 cells were determined. As a result, compounds **1**, **4**, **10**, and **15** could inhibit NO release from RAW264.7 cells in a dose-dependent manner at 10, 25, and 50 μM (Table 5; Figure 3).

**Figure 4:**
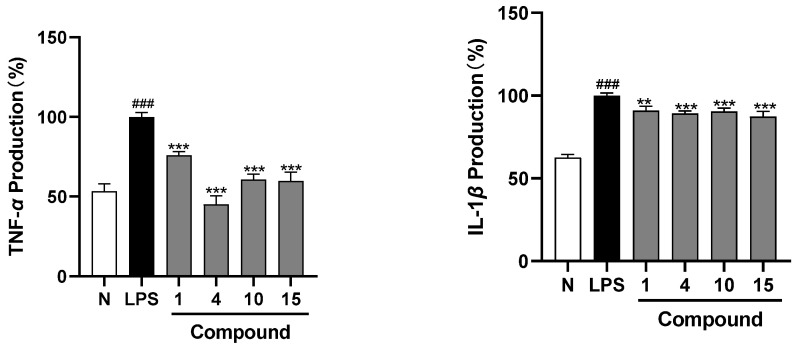
Effect of compounds **1**, **4**, **10**, and **15** on the concentration of TNF-α and IL-1β in LPS-induced RAW264.7 cells. Cells were incubated with LPS (0.5 μg/mL) with or without compounds **1**, **4**, **10**, and **15** for 18 h. The results are presented as the mean ± standard error (*n* = 3), and a *p*-value less than 0.05 was expressed as statistically significant (^###^ *p* < 0.001 vs. normal group; ** *p* < 0.01, *** *p* < 0.001 vs. LPS-treatment group).

Furthermore, an Elisa assay was applied to investigate the inhibition of active compounds **1**, **4**, **10**, and **15** on the IL-1β and TNF-α produced by LPS-induced RAW264.7 cells to verify their anti-inflammatory activities. It was found that all of them could significantly down-regulate the levels of IL-1β and TNF-α stimulated by LPS in RAW264.7 cells at 50 μM (Figure 4).

## 3. Discussion

In the Pharmacopoeia of the People’s Republic of China (hereinafter referred to as the “Pharmacopoeia”), Suo Luo Zi is recorded as the dried and mature seeds of *A. chinensis*, *A. chinensis* var. *chekiangensis*, or *A. wilsonii*. Among them, *A. chinensis* and *A. chinensis* var. *chekiangensis* have been researched more extensively. They were found to be rich in various chemical components such as escin saponins, flavonoids, organic acids, and sterols. Modern pharmacological research has shown that saponins from them showed a variety of activities such as anti-inflammatory [6], anti-edema [7], anti-viral [8], and anti-tumor [9], which have attracted the attention of many domestic and foreign experts and scholars. However, the literature research results indicated that research on the anti-inflammatory activity of *A. wilsonii* is mostly limited to escin saponins A–D, while related studies on other components are rarely reported. In our previous study, multiple saponins [10], flavonoids [11] and nitrogenous compounds [9] were proved to be its potential anti-inflammatory active components. In this article, the study of phenolic acid glycosides, terpenes, terpenoids, and organic acids was supplemented. It was found that when comparing with the other two plants, phenolic acid glycosides from the seeds of *A. wilsonii* owned a wide variety of sugar groups, including rhamnose and glucose [27,28], while phenolic acid glycosides *A. chinensis* and *A. chinensis* var. *chekiangensis* were only substituted by glucuronic acid and glucose.

The variety of compounds found in *A. wilsonii* indicate that multiple biosynthetic routes exist in this ancient plant, such as the acetate-malonate pathway, the mevalonic acid pathway, the cinnamic acid pathway, and the shikimic acid pathway [29,30,31].

While there is too little research on these compounds, among the active compounds found in this study, only 1-(2-methylbutyryl)phloroglucinyl-glucopyranoside (**10**) has been reported to exhibit potential anti-inflammatory effects, which can significantly reduce the production of inflammatory cytokine COX-1 [17]. Thus, it can be seen that more in-depth mechanism research still needs to be carried out.

## 4. Conclusions

In conclusion, four undescribed isolates, aeswilosides I–IV (**1**–**4**), and fourteen known ones were obtained from the 70% EtOH extraction of *A. wilsonii* seeds. Among the known isolates, compounds **5**, **6**, **8**–**10**, and **12**–**16** were isolated from the *Aesculus* genus for the first time; compounds **7**, **11**, **17**, and **18** were first isolated from this plant. The NMR data of **5** and **18** were reported first.

Furthermore, the NO release inhibitory effects of all the obtained compounds **1**–**18** were determined, and **1**, **4**, **10**, and **15** were found to show significant bioactivity compared with the LPS-stimulated group in RAW264.7 cells. Then, their anti-inflammatory activities were confirmed by an Elisa assay of inflammatory factors IL-1β and TNF-α.

Combining our previous research on *A. wilsonii* seeds, we can conclude that it contains a variety of chemical components, including saponins [10], flavonoids [11], nitrogenous compounds [9], and phenolic acids, which contribute to the plant’s chemical diversity. Additionally, we have discovered that all these compounds exhibit anti-inflammatory activity, indicating that *A. wilsonii* seeds possess significant potential as an inflammation inhibitor for the clinical treatment of inflammatory diseases. Furthermore, the identification of bioactive compounds from *A. wilsonii* seeds serves as a valuable reference for the development of novel anti-inflammatory agents.

## 5. Experimental Section

### 5.1. General Experimental Procedures

Optical rotations were determined using a Rudolph Autopol V automatic polarimeter. UV spectra were acquired using a Varian Cary 50 UV-Vis spectrophotometer (Varian, Inc., DE, USA). IR spectra were recorded on a Varian 640-IR FT-IR spectrophotometer (Varian, Inc.). NMR spectra were collected on Bruker Ascend 500 MHz or 600 MHz NMR spectrometers (Bruker BioSpin AG, Mass, USA). Mass spectra were obtained in the negative ion mode using a Thermo ESI-Q-Orbitrap MS spectrometer connected to an UltiMate 3000 UHPLC instrument via ESI interface (Thermo Fisher Scientific, Mass, USA). MTT and nitrite levels were determined using a BioTek Cytation 5 cell imaging multi-mode reader (BioTek, VT, USA).

Macroporous resin D101 (Haiguang Chemical Co., Ltd.), silica gel (48–75 μm, Qingdao Haiyang Chemical Co., Ltd., Qingdao, CN), YMC × Gel ODS-A-HG (50 μm, AAG12S50, YMC Co., Ltd., Kyoto, JPN), and Sephadex LH-20 (Ge Healthcare Bio-Sciences, DE, USA) were used for column chromatography (CC). Analytical HPLC was performed on Cosmosil 5C_18_-MS-II (4.6 mm i.d. × 250 mm, 5 μm, Nakalai Tesque, Inc., Kyoto, JPN) and Cosmosil PBr (4.6 mm i.d. × 250 mm, 5 μm, Nakalai Tesque, Inc.) columns. Cosmosil 5C_18_-MS-II (20 mm i.d. × 250 mm, 5 μm, Nakalai Tesque, Inc.) and Cosmosil PBr (20 mm i.d. × 250 mm, 5 μm, Nakalai Tesque, Inc.) columns were used for preparative isolation. All reagents used for phytochemical investigation were analytical grade (Concord Technology Co. Ltd., Tianjin, CN).

The RAW264.7 cell lines (Chinese Academy of Medical Science, Beijing, CN), Dulbecco’s modified eagle medium (DMEM) (Biological Industries, Kibbutz Beit Haemek, Israel) and 10% (*v*/*v*) fetal bovine serum (FBS) (Biological Industries, Israel), 100 U/mL penicillin and 100 μg/mL streptomycin (Sigma-Aldrich, St. Louis, MO, USA), dexamethasone (Sigma-Aldrich, St. Louis, MO, USA), LPS (Sigma-Aldrich, St. Louis, MO, USA), thiazolyl blue tetrazolium bromide (MTT) (Sigma-Aldrich, St. Louis, MO, USA), Mouse TNF-α ELISA kits (Biotechnology Co., Ltd., Shanghai, China), and IL-1β ELISA kits (Biotechnology Co., Ltd., Shanghai, China) were used for bioassay.

### 5.2. Plant Material

The seeds of *Aesculus wilsonii* Rehd were purchased, identified, and kept, as has been reported previously [11].

### 5.3. Extraction and Isolation

The dried seeds of *A. wilsonii* (15.0 kg) were extracted under reflux for three times (3, 2, and 2 h) using 75 L, 60 L, and 60 L of a solution containing 70% EtOH, respectively. After removal of the solvent under reduced pressure, a residue weighing 2.6 kg was obtained. The residue (2.2 kg) was loaded onto a D101 resin column, and sequentially eluted with H_2_O and 95% EtOH to yield the H_2_O (717.8 g) and 95% EtOH (SA, 847.5 g) eluates.

SA (400.0 g) was fractionated by silica gel CC [CH_2_Cl_2_-MeOH (100:0 → 100:1 → 100:3 → 100:7 → 10:1 → 8:1 → 7:1 → 5:1 → 3:1 → 1:1 → 0:100, *v*/*v*)] to produce SA1–SA14. SA8 (3.2 g) was further subjected to silica gel CC [CH_2_Cl_2_-MeOH (100:1 → 100:5 → 100:7 → 10:1 → 0:100, *v*/*v*)], and SA8-1–SA8-7 were given. SA8-3 (450.0 mg) was separated by pHPLC [MeOH-1% HAc (78:22, *v*/*v*), Cosmosil 5C18-MS-II column] to yield methyl (9*S*,10*E*,12*S*,13*S*)-9,12,13-trihydroxy-10-octadecenoate (**18**, 97.2 mg, *t*_R_ 16.7 min). SA8-5 (450.0 mg) was purified by pHPLC [MeOH-1% HAc (68:32, *v*/*v*), Cosmosil 5C18-MS-II column] to gain fulgidic acid (**16**, 22.0 mg, *t*_R_ 14.1 min) and (9*S*,10*E*,12*S*,13*S*)-9,12,13-trihydroxy-10-octadecenoic acid (**17**, 180.2 mg, *t*_R_ 16.0 min). SA9 (5.0 g) was loaded onto ODS CC [MeOH-H_2_O (20:80 → 30:70 → 40:60 → 50:50 → 60:40 → 70:30 → 80:20 → 100:0, *v*/*v*)] to give SA9-1–SA 9-14. SA9-6 (120.0 mg) was purified by pHPLC [MeOH-1% HAc (60:40, *v*/*v*), Cosmosil PBr column] to produce 1-[(2-methylpropanoyl)phloroglucinyl]-β-d-glucopyranoside (**9**, 8.8 mg, *t*_R_ 17.0 min). SA9-7 (350.0 mg) was isolated by pHPLC [MeOH-1% HAc (40:60, *v*/*v*), Cosmosil 5C18-MS-II column] to gain SA9-7-1–SA9-7-5. Among them, SA9-7-5 (25.0 mg) was elucidated to be myrseguinoside A (**13**, 25.0 mg, *t*_R_ 31.3 min). SA9-7-2 (69.8 mg) was separated by pHPLC [CH_3_CN-1% HAc (20:80, *v*/*v*), Cosmosil 5C18-MS-II column], and (–)-pinoresinol 4-*O*-β-d-glucoside) (**11**, 30.3 mg, *t*_R_ 26.6 min) was yielded. SA9-9 (1000.0 mg) was purified by pHPLC with MeOH-1% HAc (65:35, *v*/*v*, Cosmosil PBr column) and CH_3_CN-1% HAc (31:69, *v*/*v*, Cosmosil 5C18-MS-II column) to obtain 1-(2-methylbutyryl)phloroglucinyl-glucopyranoside (**10**, 13.5 mg, *t*_R_ 17.6 min). SA10 (4.8 g) was fractionated by ODS CC [MeOH-H_2_O (20:80 → 40:60 → 45:55 → 50:50 → 55:45 → 60:40 → 100:0, *v*/*v*)] to yield SA10-1–SA10-13. SA10-3 (1000.0 mg) was subjected to pHPLC [MeOH-1% HAc (30:70, *v*/*v*), Cosmosil 5C18-MS-II column] to obtain SA10-3-1–SA10-3-11. SA10-3-3 (18.5 mg) was further purified by pHPLC [CH_3_CN-1% HAc (14:86, *v*/*v*), Cosmosil 5C18-MS-II column] to give lippianoside E (**14**, 9.6 mg, *t*_R_ 23.8 min). SA10-3-10 (35.3 mg) was separated by pHPLC [CH_3_CN-1% HAc (16:84, *v*/*v*), Cosmosil 5C18-MS-II column], to produce 2-phenethyl-*O*-α-l-rhamnopyranosyl(1→6)-β-d-glucopyranoside (**7**, 13.7 mg, *t*_R_ 30.7 min). SA10-5 (500.0 mg) was loaded onto pHPLC [CH_3_CN-1% HAc (20:80, *v*/*v*), Cosmosil 5C18-MS-II column] to obtain SA10-5-1–SA10-5-10. SA10-5-4 (11.5 mg) and SA10-5-6 (34.7 mg) were purified by pHPLC [MeOH-1% HAc (43:57, *v*/*v*), Cosmosil 5C18-MS-II column] to gain aeswiloside IV (**4**, 3.0 mg, *t*_R_ 41.6 min) and pisuminic acid (**15**, 12.7 mg, *t*_R_ 47.1 min), respectively. SA 10-6 (100.0 mg) was isolated by pHPLC [CH_3_CN-1% HAc (23:77, *v*/*v*), Cosmosil 5C18-MS-II column] to produce 3-*O*-[α-l-arabinopyranosyl(1→6)-β-d-glucopyranosyl]oct-1-ene-3-ol (**12**, 9.2 mg, *t*_R_ 31.3 min). SA11 (30.0 g) was subjected to MCI gel CHP 20P CC [MeOH-H_2_O (20:80 → 30:70 → 40:60 → 50:50 → 60:40 → 70:30 → 80:20 → 100:0, *v*/*v*)] to obtain SA11-1–SA11-19. SA11-4 (200.0 mg) was separated by pHPLC [MeOH-1% HAc (31:69, *v*/*v*), Cosmosil 5C18-MS-II column] to produce aeswiloside I (**1**, 16.0 mg, *t*_R_ 11.4 min), asechipuroside A (**8**, 12.8 mg, *t*_R_ 14.5 min), phenyl-*O*-α-l-rhamnopyranosyl(1→6)-β-d-glucopyranoside (**5**, 12.9 mg, *t*_R_ 16.8 min), and aeswiloside III (**3**, 9.1 mg, *t*_R_ 25.6 min). SA11-5 (150.0 mg) was purified by pHPLC [CH_3_CN-1% HAc (11:89, *v*/*v*), Cosmosil 5C18-MS-II column] to obtain aeswiloside II (**2**, 10.1 mg, *t*_R_ 23.2 min). SA11-8 (300.0 mg) was purified by pHPLC [CH_3_CN-1% HAc (13:87, *v*/*v*), Cosmosil 5C_18_-MS-II column] to give benzyl-*O*-α-l-rhamnopyranosyl(1→6)-β-d-glucopyranoside (**6**, 22.7 mg, *t*_R_ 31.7 min).

### 5.4. Spectral Data of ***1**–**18***

#### 5.4.1. Aeswiloside I (**1**)

White powder; [α]_D_^25^ −36.4 (*conc*. 0.044, MeOH); UV *λ*_max_ (MeOH) nm (log *ε*): 221 (4.29), 264 (4.02), 296 (3.77); IR *ν*_max_ (KBr) cm^−1^: 3362, 2926, 1703, 1599, 1519, 1457, 1285, 1217, 1127, 1067; ^1^H NMR (CD_3_OD, 600 MHz) and ^13^C NMR (CD_3_OD, 150 MHz) data: see Table 1; ESI-Q-Orbitrap MS: *m*/*z* 475.14618 [M − H]^−^ (calcd. for C_20_H_27_O_13_, 475.14461).

#### 5.4.2. Aeswiloside II (**2**)

White powder; [α]_D_^25^ −57.1 (*conc*. 0.035, MeOH); UV λ_max_ (MeOH) nm (log ε): 231 (3.67), 282 (3.23); IR ν_max_ (KBr) cm^−1^: 3368, 2927, 1703, 1606, 1560, 1518, 1455, 1277, 1161, 1126, 1067, 1046; ^1^H NMR (CD_3_OD, 500 MHz) and ^13^C NMR (CD_3_OD, 125 MHz) data: see Table 2; ESI-Q-Orbitrap MS: m/z 461.16696 [M − H]^−^ (calcd. for C_20_H_29_O_12_, 461.16535).

#### 5.4.3. Aeswiloside III (**3**)

White powder; [α]_D_^25^ −50.0 (*conc*. 0.032, MeOH); UV *λ*_max_ (MeOH) nm (log *ε*): 284 (2.96); IR *ν*_max_ (KBr) cm^−1^: 3363, 2924, 1658, 1067, 1048; ^1^H NMR (CD_3_OD, 600 MHz) and ^13^C NMR (CD_3_OD, 150 MHz) data: see Table 3; ESI-Q-Orbitrap MS: *m*/*z* 439.18240 [M + COOH]^−^ (calcd for C_18_H_31_O_12_, 439.18100).

#### 5.4.4. Aeswiloside IV (**4**)

White powder; [α]_D_^25^ −14.1 (*conc*. 0.043, MeOH); IR *ν*_max_ (KBr) cm^−1^: 3352, 2973, 2926, 2849, 1450, 1164, 1077, 1017, 923; ^1^H NMR (CD_3_OD, 500 MHz) and ^13^C NMR (CD_3_OD, 125 MHz) data: see Table 4; ESI-Q-Orbitrap MS: *m*/*z* 479.25037 [M + COOH]^−^ (calcd. for C_22_H_39_O_11_, 479.24869).

#### 5.4.5. Phenyl-*O*-α-l-Rhamnopyranosyl(1→6)-β-d-Glucopyranoside (**5**)

White powder; ^1^H NMR (CD_3_OD, 600 MHz) δ_H_: 7.08 (2H, dd, *J* = 1.2, 7.2 Hz, H-2,6), 7.29 (2H, dt, *J* = 1.2, 7.2 Hz, H-3,5), 7.00 (1H, tt, *J* = 1.2, 7.2 Hz, H-4), 4.85 (1H, d, *J* = 8.4 Hz, H-1′), 3.46 (1H, dd, *J* = 8.4, 9.0 Hz, H-2′), 3.44 (1H, dd, *J* = 9.0, 9.0 Hz, H-3′), 3.37 (1H, dd, *J* = 9.6, 9.6 Hz, H-4′), 3.55 (1H, m, H-5′), [3.60 (1H, dd, *J* = 6.6, 11.4 Hz), 4.02 (1H, dd, *J* = 1.8, 11.4 Hz), H_2_-6′], 4.70 (1H, d, *J* = 1.8 Hz, H-1″), 3.83 (1H, dd, *J* = 1.8, 3.0 Hz, H-2″), 3.70 (1H, dd, *J* = 3.0, 9.6 Hz, H-3″), 3.36 (1H, dd, *J* = 9.6, 9.6 Hz, H-4″), 3.65 (1H, m, H-5″), 1.20 (3H, d, *J* = 6.0 Hz, H_3_-6″); ^13^C NMR (CD_3_OD, 150 MHz) δ_C_: 159.1 (C-1), 117.9 (C-2,6), 130.5 (C-3,5), 123.5 (C-4), 102.4 (C-1′), 75.0 (C-2′), 78.1 (C-3′), 71.6 (C-4′), 77.0 (C-5′), 67.9 (C-6′), 102.2 (C-1″), 72.2 (C-2″), 72.5 (C-3″), 74.1 (C-4″), 69.9 (C-5″), 18.0 (C-6″); ESI-Q-Orbitrap MS: *m*/*z* 447.15134 [M + COOH]^−^ (calcd. for C_19_H_27_O_12_, 447.14970).

#### 5.4.6. Benzyl-*O*-α-l-Rhamnopyranosyl(1→6)-β-d-Glucopyranoside (**6**)

White powder; ^1^H NMR (CD_3_OD, 600 MHz) δ_H_: 7.41 (2H, br. d, ca. *J* = 7 Hz, H-2,6), 7.33 (2H, t like, ca. *J* = 7 Hz, H-3,5), 7.27 (1H, t like, *J* = 7 Hz, H-4), [4.64 (1H, d, *J* = 11.4 Hz), 4.88 (1H, d, *J* = 11.4 Hz), H_2_-7], 4.32 (1H, d, *J* = 7.8 Hz, H-1′), 3.24 (1H, dd, *J* = 7.8, 9.0 Hz, H-2′), 3.34 (1H, dd, *J* = 9.0, 9.0 Hz, H-3′), 3.29 (1H, dd, *J* = 9.0, 9.0 Hz, H-4′), 3.38 (1H, m, H-5′), [3.64 (1H, dd, *J* = 6.0, 11.4 Hz), 4.00 (1H, dd, *J* = 1.8, 10.8 Hz), H_2_-6′], 4.78 (1H, d, *J* = 1.2 Hz, H-1″), 3.87 (1H, dd, *J* = 1.2, 3.6 Hz, H-2″), 3.70 (1H, dd, *J* = 3.6, 9.6 Hz, H-3″), 3.38 (1H, *J* = 9.6, 9.6 Hz, H-4″), 3.69 (1H, m, H-5″), 1.27 (3H, d, *J* = 6.0 Hz, H_3_-6″); ^13^C NMR (CD_3_OD, 150 MHz) δ_C_: 138.9 (C-1), 129.4 (C-2,6, 3,5), 128.8 (C-4), 71.8 (C-7), 103.2 (C-1′), 75.1 (C-2′), 78.1 (C-3′), 71.8 (C-4′), 77.0 (C-5′), 68.2 (C-6′), 102.3 (C-1″), 72.3 (C-2″), 72.4 (C-3″), 74.1 (C-4″), 69.9 (C-5″), 18.1 (C-6″); ESI-Q-Orbitrap MS: *m*/*z* 461.16733 [M + COOH]^−^ (calcd. For C_20_H_29_O_12_, 461.16535).

#### 5.4.7. 2-Phenethyl-*O*-α-l-Rhamnopyranosyl(1→6)-β-d-Glucopyranoside (**7**)

White powder; ^1^H NMR (DMSO-*d*_6_, 500 MHz) δ_H_: 7.27 (2H, m, H-2,6), 7.28 (2H, m, H-3,5), 7.27 (1H, t like, ca. *J* = 8 Hz, H-4), 2.86 (2H, t, *J* = 7.0 Hz, H-7), [3.68 (1H, q like, ca. *J* = 8 Hz), 3.89 (1H, q like, ca. *J* = 8 Hz), H_2_-8], 4.19 (1H, d, *J* = 7.5 Hz, H-1′), 2.96 (1H, dd, *J* = 7.5, 8.5 Hz, H-2′), 3.14 (1H, dd, *J* = 8.5, 9.0 Hz, H-3′), 3.00 (1H, dd, *J* = 9.0, 9.0 Hz, H-4′), 3.27 (1H, m, H-5′), [3.43 (1H, dd, *J* = 6.5, 11.0 Hz), 3.82 (1H, br. D, ca. *J* = 11 Hz), H_2_-6′], 4.60 (1H, br. S, H-1″), 3.61 (1H, br. D, ca. *J* = 4 Hz, H-2″), 3.42 (1H, dd, *J* = 3.5, 9.5 Hz, H-3″), 3.17 (1H, dd, *J* = 9.5, 9.5 Hz, H-4″), 3.45 (1H, m, H-5″), 1.13 (3H, d, *J* = 6.5 Hz, H_3_-6″); ^13^C NMR (DMSO-*d*_6_, 125 MHz) δ_C_: 138.6 (C-1), 128.8 (C-2,6), 128.1 (C-3,5), 125.9 (C-4), 35.6 (C-7), 69.4 (C-8), 102.8 (C-1′), 73.2 (C-2′), 76.5 (C-3′), 70.1 (C-4′), 75.3 (C-5′), 66.9 (C-6′), 100.7 (C-1″), 70.4 (C-2″), 70.6 (C-3″), 71.8 (C-4″), 68.2 (C-5″), 17.8 (C-6″); ESI-Q-Orbitrap MS: *m*/*z* 429.17685 [M − H]^−^ (calcd. for C_20_H_29_O_10_, 429.17552).

#### 5.4.8. Asechipuroside A (**8**)

White powder; ^1^H NMR (CD_3_OD, 600 MHz) δ_H_: 7.06 (2H, d, *J* = 8.0 Hz, H-2,6), 6.69 (2H, d, *J* = 8.0 Hz, H-3,5), 2.83 (2H, m, H_2_-7), [3.68 (1H, m), 3.96 (1H, m), H_2_-8], 4.28 (1H, d, *J* = 7.8 Hz, H-1′), 3.17 (1H, dd, *J* = 7.8, 9.0 Hz, H-2′), 3.33 (1H, dd, *J* = 9.0, 9.0 Hz, H-3′), 3.27 (1H, dd, *J* = 9.0, 9.6 Hz, H-4′), 3.37 (1H, m, H-5′), [3.61 (1H, dd, *J* = 6.0, 11.4 Hz), 3.97 (1H, m), H_2_-6′], 4.74 (1H, d, *J* = 1.8 Hz, H-1″), 3.83 (1H, dd, *J* = 1.8, 3.6 Hz, H-2″), 3.67 (1H, dd, *J* = 3.6, 9.0 Hz, H-3″), 3.36 (1H, dd, *J* = 9.0, 9.0 Hz, H-4″), 3.66 (1H, m, H-5″), 1.25 (3H, d, *J* = 6.0 Hz, H_3_-6″); ^13^C NMR (CD_3_OD, 150 MHz) δ_C_: 130.7 (C-1), 131.0 (C-2,6), 116.2 (C-3,5), 156.8 (C-4), 36.5 (C-7), 72.3 (C-8), 104.5 (C-1′), 75.1 (C-2′), 78.1 (C-3′), 71.7 (C-4′), 76.9 (C-5′), 68.1 (C-6′), 102.3 (C-1″), 72.2 (C-2″), 72.4 (C-3″), 74.1 (C-4″), 69.8 (C-5″), 18.1 (C-6″); ESI-Q-Orbitrap MS: *m*/*z* 445.17215 [M − H]^−^ (calcd. For C_20_H_29_O_11_, 445.17044).

#### 5.4.9. 1-[(2-Methylpropanoyl)Phloroglucinyl]-β-d-Glucopyranoside (**9**)

White powder; ^1^H NMR (CD_3_OD, 600 MHz) δ_H_: 5.95 (1H, d, *J* = 2.4 Hz, H-4), 6.18 (1H, d, *J* = 2.4 Hz, H-6), 3.99 (1H, sept, *J* = 6.6 Hz, H-2′), 1.15 (3H, d, *J* = 6.6 Hz, H_3_-3′), 1.13 (3H, d, *J* = 6.6 Hz, H_3_-4′), 5.04 (1H, d, *J* = 7.8 Hz, H-1″), 3.50 (1H, dd, *J* = 7.8, 9.0 Hz, H-2″), 3.47 (1H, dd, *J* = 9.0, 9.0 Hz, H-3″), 3.39 (1H, dd, *J* = 9.0, 9.6 Hz, H-4″), 3.46 (1H, m, H-5″), [3.72 (1H, dd, *J* = 6.0, 12.0 Hz), 3.91 (1H, dd, *J* = 1.8, 12.0 Hz), H_2_-6″]; ^13^C NMR (CD_3_OD, 150 MHz) δ_C_: 161.7 (C-1), 106.3 (C-2), 167.5 (C-3), 98.3 (C-4), 165.6 (C-5), 95.4 (C-6), 212.0 (C-1′), 40.5 (C-2′), 20.3 (C-3′), 19.5 (C-4′), 101.6 (C-1″), 74.9 (C-2″), 78.7 (C-3″), 71.2 (C-4″), 78.4 (C-5″), 62.5 (C-6″); ESI-Q-Orbitrap MS: *m*/*z* 357.11917 [M − H]^−^ (calcd. for C_16_H_21_O_9_ 357.11801).

#### 5.4.10. 1-(2-Methylbutyryl)Phloroglucinyl-Glucopyranoside (**10**)

White powder; CD (*conc*. 0.0027 M, MeOH) mdeg (*λ*nm): +0.98 (271), −4.20 (304); ^1^H NMR (CD_3_OD, 600 MHz) δ_H_: 5.96 (1H, d, *J* = 2.4 Hz, H-4), 6.19 (1H, d, *J* = 2.4 Hz, H-6), 3.90 (1H, sex, *J* = 6.6 Hz, H-2′), 1.38, 1.80 (1H each, both m, H_2_-3′), 0.89 (3H, t, *J* = 7.8 Hz, H_3_-4′), 1.13 (3H, d, *J* = 6.6 Hz, H_3_-5′), 5.03 (1H, d, *J* = 7.2 Hz, H-1″), 3.52 (1H, dd, *J* = 7.2, 9.0 Hz, H-2″), 3.46 (1H, dd, *J* = 8.4, 9.0 Hz, H-3″), 3.39 (1H, dd, *J* = 8.4, 9.0 Hz, H-4″), 3.46 (1H, m, H-5″), [3.71 (1H, dd, *J* = 5.4, 12.0 Hz), 3.92 (1H, dd, *J* = 1.8, 12.0 Hz), H_2_-6″]; ^13^C NMR (CD_3_OD, 150 MHz) δ_C_: 161.8 (C-1), 106.9 (C-2), 167.5 (C-3), 98.4 (C-4), 165.6 (C-5), 95.4 (C-6), 211.8 (C-1′), 47.0 (C-2′), 28.3 (C-3′), 12.1 (C-4′), 16.9 (C-5′), 101.7 (C-1″), 74.8 (C-2″), 78.8 (C-3″), 71.3 (C-4″), 78.5 (C-5″), 62.5 (C-6″); ESI-Q-Orbitrap MS: *m*/*z* 371.13489 [M − H]^−^ (calcd. For C_17_H_23_O_9_, 371.13365).

#### 5.4.11. (−)-Pinoresinol 4-O-β-d-Glucoside (**11**)

White powder; CD (*conc*. 0.0001 M, MeOH) mdeg (*λ*_nm_): −11.92 (200), −0.94 (229); ^1^H NMR (CD_3_OD, 500 MHz) δ_H_: 7.01 (1H, d, *J* = 1.5 Hz, H-2), 7.13 (1H, d, *J* = 8.5 Hz, H-5), 6.89 (1H, dd, *J* = 1.5, 8.5 Hz, H-6), 4.73 (1H, d, *J* = 3.5 Hz, H-7), 3.09 (1H, m, H-8), 3.84, 4.21 (1H each, both m, H_2_-9), 6.93 (1H, d, *J* = 1.5 Hz, H-2′), 6.76 (1H, d, *J* = 8.5 Hz, H-5′), 6.78 (1H, dd, *J* = 1.5, 8.5 Hz, H-6′), 4.68 (1H, d, *J* = 4.5 Hz, H-7′), 3.09 (1H, m, H-8′), 3.84, 4.21 (1H each, both m, H_2_-9′), 4.87 (1H, d, *J* = 7.0 Hz, H-1″), 3.49 (1H, dd, *J* = 7.0, 9.5 Hz, H-2″), 3.47 (1H, dd, *J* = 9.5, 9.5 Hz, H-3″), 3.39 (1H, m, overlapped, H-4″), 3.39 (1H, m, overlapped, H-5″), [3.68 (1H, dd, *J* = 4.5, 11.0 Hz), 3.86 (1H, br. D, ca. *J* = 11 Hz), H_2_-6″], 3.85, 3.84 (3H each, both s, 3, 3′-OCH_3_); ^13^C NMR (CD_3_OD, 125 MHz) δ_C_: 137.5 (C-1), 111.7 (C-2), 151.0 (C-3), 147.5 (C-4), 118.0 (C-5), 119.8 (C-6), 87.1 (C-7), 55.5 (C-8), 72.7 (C-9), 133.8 (C-1′), 111.0 (C-2′), 149.1 (C-3′), 147.3 (C-4′), 116.1 (C-5′), 120.1 (C-6′), 87.5 (C-7′), 55.3 (C-8′), 72.7 (C-9′), 102.8 (C-1″), 74.9 (C-2″), 77.8 (C-3″), 71.4 (C-4″), 78.2 (C-5″), 62.5 (C-6″), 56.8 (3-OCH_3_), 56.5 (3′-OCH_3_); ESI-Q-Orbitrap MS: *m*/*z* 519.18768 [M − H]^−^ (calcd. For C_26_H_31_O_11_, 520.19391).

#### 5.4.12. 3-*O*-[α-l-Arabinopyranosyl(1→6)-β-d-Glucopyranosyl]oct-1-ene-3-ol (**12**)

Colorless oil; [α]_D_^25^ −23.5 (*conc*. 0.026, MeOH); ^1^H NMR (C_5_D_5_N, 600 MHz) δ_H_: [5.20 (1H, d, *J* = 10.8 Hz), 5.44 (1H, d, *J* = 16.8 Hz), H_2_-1], 6.13 (1H, m, H-2), 4.48 (1H, m, H-3), 1.64, 1.77 (1H each, both m, H_2_-4), 1.46 (2H, m, H_2_-5), 1.18 (4H, m, H_2_-6 and 7), 4.90 (1H, d, *J* = 7.8 Hz, H-1′), 4.00 (1H, dd, *J* = 7.8, 8.4 Hz, H-2′), 4.20 (1H, dd, *J* = 8.4, 9.0 Hz, H-3′), 4.14 (1H, dd, *J* = 9.0, 9.6 Hz, H-4′), 4.05 (1H, m, H-5′), [4.30 (1H, dd, *J* = 6.0, 12.0 Hz), 4.77 (1H, br. d, ca. *J* = 12 Hz), H_2_-6′], 4.99 (1H, d, *J* = 6.0 Hz, H-1″), 4.46 (1H, dd, *J* = 6.0, 7.8 Hz, H-2″), 4.19 (1H, dd, *J* = 7.8, 9.0 Hz, H-3″), 4.34 (1H, m, H-4″), [3.76 (1H, br. d, ca. *J* = 12.0 Hz), 4.31 (1H, dd, *J* = 5.4, 12.0 Hz), H_2_-5″]; ^13^C NMR (C_5_D_5_N, 150 MHz) δ_C_: 115.3 (C-1), 140.8 (C-2), 80.9 (C-3), 35.1 (C-4), 24.8 (C-5), 32.2 (C-6), 22.8 (C-7), 14.2 (C-8), 103.6 (C-1′), 75.3 (C-2′), 78.6 (C-3′), 71.9 (C-4′), 77.1 (C-5′), 69.5 (C-6′), 105.3 (C-1″), 72.4 (C-2″), 74.4 (C-3″), 69.1 (C-4″), 66.4 (C-5″); ESI-Q-Orbitrap MS: *m*/*z* 467.21439 [M + COOH]− (calcd. for C_20_H_35_O_12_, 467.21230).

#### 5.4.13. Myrseguinoside A (**13**)

White powder; ^1^H NMR (CD_3_OD, 500 MHz) δ_H_: 5.63 (1H, br. d, ca. *J* = 5 Hz, H-2), 1.77, 2.13 (1H each, both m, H_2_-3), 1.88 (1H, m, H-4), [1.25 (1H, dt like, ca. *J* = 4, 14 Hz), 2.22 (1H, m), H_2_-5], 1.79 (3H, br. s, H_3_-7), 1.20 (3H, s, H_3_-9), 1.16 (3H, s, H_3_-10), 4.37 (1H, d, *J* = 8.0 Hz, H-1′), 3.19 (1H, dd, *J* = 8.0, 8.5 Hz, H-2′), 3.39 (1H, dd, *J* = 8.5, 8.5 Hz, H-3′), 3.32 (1H, dd, *J* = 8.5, 8.5 Hz, H-4′), 3.30 (1H, m, H-5′), [3.70 (1H, dd, *J* = 5.0, 11.5 Hz), 3.90 (1H, dd, *J* = 1.5, 11.5 Hz), H_2_-6′]; ^13^C NMR (CD_3_OD, 125 MHz) δ_C_: 134.1 (C-1), 127.4 (C-2), 28.3 (C-3), 39.7 (C-4), 29.9 (C-5), 76.0 (C-6), 21.1 (C-7), 73.0 (C-8), 27.8 (C-9), 26.1 (C-10), 102.2 (C-1′), 75.1 (C-2′), 78.2 (C-3′), 71.9 (C-4′), 78.0 (C-5′), 63.0 (C-6′); ESI-Q-Orbitrap MS: *m*/*z* 377.18222 [M + COOH]^−^ (calcd. for C_17_H_29_O_9_, 377.18061).

#### 5.4.14. Lippianoside E (**14**)

White powder; ^1^H NMR (DMSO-*d*_6_, 500 MHz) δ_H_: [1.20 (1H, dd, *J* = 11.5, 11.5 Hz), 1.80 (1H, ddd, *J* = 3.0, 4.0, 11.5 Hz), H_2_-2], 4.15 (1H, m, H-3), [1.18 (1H, dd, *J* = 11.5, 11.5 Hz), 2.38 (1H, ddd, *J* = 3.0, 4.0, 11.5 Hz), H_2_-4], 5.86 (1H, s, H-7), 2.12 (3H, s, H_3_-10), 1.28 (3H, s, H_3_-11), 1.05 (3H, s, H_3_-12), 1.33 (3H, s, H_3_-13), 4.36 (1H, d, *J* = 7.8 Hz, H-1′), 2.91 (1H, dd, *J* = 7.5, 8.5 Hz, H-2′), 3.15 (1H, dd, *J* = 8.5, 8.5 Hz, H-3′), 3.00 (1H, dd, *J* = 8.5, 9.0 Hz, H-4′), 3.06 (1H, m, H-5′), [3.37 (1H, dd, *J* = 6.0, 11.5 Hz), 3.62 (1H, br. d, ca. *J* = 11 Hz), H_2_-6′]; ^13^C NMR (DMSO-*d*_6_, 125 MHz) δ_C_: 35.4 (C-1), 49.1 (C-2), 61.0 (C-3), 45.9 (C-4), 76.9 (C-5), 117.4 (C-6), 99.8 (C-7), 210.6 (C-8), 197.5 (C-9), 26.2 (C-10), 29.0 (C-11), 31.7 (C-12), 96.8 (C-1′), 73.6 (C-2′), 77.2 (C-3′), 70.1 (C-4′), 76.6 (C-5′), 61.1 (C-6′); ESI-Q-Orbitrap MS: *m*/*z* 425.17201 [M + Na]^+^ (calcd. for C_19_H_30_O_9_Na, 425.17820).

#### 5.4.15. Pisuminic Acid (**15**)

White powder; [α]_D_^25^ −37.7 (*conc*. 0.027, MeOH); ^1^H NMR (C_5_D_5_N, 600 MHz) δ_H_: [1.01 (1H, dt, *J* = 3.0, 13.2 Hz), 1.87 (1H, m, overlapped), H_2_-1], [1.50 (1H, br. D, ca. *J* = 14 Hz), 2.23 (1H, m, overlapped), H_2_-2], [1.24 (1H, dt, *J* = 4.2, 13.8 Hz), 2.51 (1H, br. D, ca. *J* = 13 Hz), H_2_-3], 2.41 (1H, d, *J* = 10.8 Hz, H-5), 4.97 (1H, br. D, ca. *J* = 11 Hz, H-6), 4.18 (1H, m, H-7), 1.83 (1H, br. D, ca. *J* = 8 Hz, H-9), 1.60, 1.68 (1H each, both m, H_2_-11), 1.55, 1.92 (1H each, both m, H_2_-12), [2.12 (1H, d, *J* = 14.4 Hz), 2.59 (1H, d, *J* = 14.4 Hz), H_2_-14], [1.99 (1H, d, *J* = 11.4 Hz), 2.23 (1H, m, overlapped), H_2_-15], 2.48 (1H, m, H-16), [4.10 (1H, d, *J* = 10.8 Hz), 4.17 (1H, d, *J* = 10.8 Hz), H_2_-17], 1.87 (3H, s, H_3_-18), 1.28 (3H, s, H_3_-20); ^13^C NMR (C_5_D_5_N, 150 MHz) δ_C_: 41.1 (C-1), 19.8 (C-2), 40.6 (C-3), 44.6 (C-4), 52.0 (C-5), 72.0 (C-6), 82.6 (C-7), 49.4 (C-8), 50.0 (C-9), 41.1 (C-10), 18.7 (C-11), 27.4 (C-12), 81.3 (C-13), 51.0 (C-14), 36.9 (C-15), 45.8 (C-16), 66.5 (C-17), 33.3 (C-18), 181.3 (C-19), 17.2 (C-20). ESI-Q-Orbitrap MS: *m*/*z* 367.21310 [M − H]^−^ (calcd. For C_20_H_31_O_6_, 367.21152).

#### 5.4.16. Fulgidic Acid (**16**)

White powder; [α]_D_^25^ −14.8 (*conc*. 0.054, MeOH); ^1^H NMR (CD_3_OD, 500 MHz) δ_H_: 2.27 (2H, t, *J* = 7.5 Hz, H_2_-2), 1.59 (2H, m, H_2_-3), 1.27–1.45 (8H, H_2_-4–7), 1.52 (2H, H_2_-8), 4.05 (1H, q like, ca. *J* = 6 Hz, H-9), 5.71 (1H, dd, *J* = 5.0, 15.5 Hz, H-10), 5.69 (1H, dd, *J* = 5.0, 15.5 Hz, H-11), 3.95 (1H, t like, ca. *J* = 5 Hz, H-12), 3.45 (1H, m, ca. *J* = 5 Hz, H-13), [2.11 (1H, m), 2.34 (1H, ddd, *J* = 5.5, 5.5, 14.5 Hz), H_2_-14], 5.43 (1H, dd, *J* = 6.0, 11.0 Hz, H-15), 5.47 (1H, dd, *J* = 6.5, 11.0 Hz), H-16], 2.06 (2H, m, H_2_-17), 0.96 (3H, t, *J* = 7.5 Hz, H_3_-18); ^13^C NMR (CD_3_OD, 125 MHz) δ_C_: 177.7 (C-1), 35.0 (C-2), 26.1 (C-3), 30.2 (C-4), 30.4 (C-5), 30.6 (C-6), 26.5 (C-7), 38.4 (C-8), 73.1 (C-9), 136.6 (C-10), 131.1 (C-11), 75.8 (C-12), 75.9 (C-13), 31.6 (C-14), 126.4 (C-15), 134.4 (C-16), 21.7 (C-17), 14.6 (C-18); ESI-Q-Orbitrap MS: *m*/*z* 327.21808 [M − H]^−^ (calcd. for C_18_H_31_O_5_, 327.21660).

#### 5.4.17. (9*S*,10*E*,12*S*,13*S*)-9,12,13-Trihydroxy-10-Octadecenoic Acid (**17**)

White powder; [α]_D_^25^ −19.5 (*conc*. 0.021, MeOH); ^1^H NMR (CD_3_OD, 500 MHz) δ_H_: 2.27 (2H, t, *J* = 7.5 Hz, H_2_-2), 1.59 (2H, m, H_2_-3), 1.32–1.52 (18H, H_2_-4–8 and 14–17), 4.05 (1H, q like, ca. *J* = 6 Hz, H-9), 5.72 (1H, dd, *J* = 5.5, 15.5 Hz, H-10), 5.67 (1H, dd, *J* = 5.5, 15.5 Hz, H-11), 3.90 (1H, t like, ca. *J* = 6 Hz, H-12), 3.42 (1H, m, H-13), 0.90 (3H, t, *J* = 7.0 Hz, H_3_-18); ^13^C NMR (CD_3_OD, 125 MHz) δ_C_: 177.6 (C-1), 34.9 (C-2), 26.0 (C-3), 30.1 (C-4), 30.3 (C-5), 30.5 (C-6), 26.5 (C-7), 38.3 (C-8), 73.0 (C-9), 136.5 (C-10), 131.0 (C-11), 76.5 (C-12), 75.7 (C-13), 33.5 (C-14), 26.4 (C-15), 33.1 (C-16), 23.7 (C-17), 14.4 (C-18); ESI-Q-Orbitrap MS: *m*/*z* 329.23392 [M − H]^−^ (calcd. For C_18_H_33_O_5_, 329.23225).

#### 5.4.18. Methyl (9*S*,10*E*,12*S*,13*S*)-9,12,13-Trihydroxy-10-Octadecenoate (**18**)

White powder; [α]_D_^25^ −13.5 (*conc*. 0.103, MeOH); ^1^H NMR (CD_3_OD, 500 MHz) δ_H_: 2.31 (2H, t, *J* = 7.5 Hz, H_2_-2), 1.59 (2H, m, H_2_-3), 1.32–1.52 (18H, H_2_-4–8 and 14–17), 4.05 (1H, q like, ca. *J* = 6 Hz, H-9), 5.72 (1H, dd, *J* = 5.0, 15.5 Hz, H-10), 5.67 (1H, dd, *J* = 5.5, 15.5 Hz, H-11), 3.90 (1H, t like, ca. *J* = 6 Hz, H-12), 3.41 (1H, m, H-13), 0.90 (3H, t, *J* = 7.0 Hz, H_3_-18), 3.64 (3H, s, 1-COO*CH_3_*); ^13^C NMR (CD_3_OD, 125 MHz) δ_C_: 175.9 (C-1), 34.8 (C-2), 26.0 (C-3), 30.1 (C-4), 30.4 (C-5), 30.6 (C-6), 26.5 (C-7), 38.3 (C-8), 73.0 (C-9), 136.5 (C-10), 131.1 (C-11), 76.5 (C-12), 75.8 (C-13), 33.5 (C-14), 26.6 (C-15), 33.1 (C-16), 23.7 (C-17), 14.5 (C-18), 52.0 (1-COO*CH_3_*); ESI-Q-Orbitrap MS: *m*/*z* 367.24493 [M + Na]^+^ (calcd. for C_19_H_36_O_5_Na, 367.24550).

### 5.5. Acid Hydrolysis of Compounds ***1**–**4***

Compounds **1**–**4** (1.5 mg each) dissolved in 2.0 mL 1 M HCl were heated under reflux for 3 h, respectively. The reaction products were partitioned by EtOAc (3 × 2.0 mL). The aqueous layer extracts were analyzed using HPLC with a Kaseisorb LC NH_2_-60-5 column (4.6 mm i.d. × 250 mm, Tokyo Kasei Co., Ltd., Tokyo, Japan). The mobile phase consisted of CH_3_CN-H_2_O (80:20, *v*/*v*), and the flow rate was set at 0.7 mL/min. By comparing the retention time and optical rotation with those of d-glucose and l-rhamnose standards, the presence of d-glucose (*t*_R_: 10.2 min, positive optical rotation) and l-rhamnose (*t*_R_: 7.4 min, negative optical rotation) were clarified.

### 5.6. Scifinder Searching

The novelty of compounds **1**–**4** was determined by searching their SMILES codes in SciFinder in an absolute retrieval mode. Their SMILES codes are listed as follows.

Aeswiloside I (**1**): O[C@@H]1[C@@H](O)[C@H](O)[C@@H](CO[C@H]2[C@H](O)[C@H](O)[C@@H](O)[C@H](C)O2)O[C@H]1OC(C3=CC(OC)=C(O)C=C3)=O

Aeswiloside II (**2**): O[C@@H]1[C@@H](O)[C@H](O)[C@@H](CO[C@H]2[C@H](O)[C@H](O)[C@@H](O)[C@H](C)O2)O[C@H]1OCC3=CC(OC)=C(O)C=C3

Aeswiloside III (**3**): C/C(C)=C\CO[C@@H]1O[C@H](CO[C@H]2[C@H](O)[C@H](O)[C@@H](O)[C@H](C)O2)[C@@H](O)[C@H](O)[C@H]1O

Aeswiloside IV (**4**): O[C@@H]1[C@@H](O)[C@H](O)[C@@H](CO)O[C@H]1OC(C(C)(O)C=C)C/C=C(C)/CCC(C(C)(O)C)O

### 5.7. Bioassays

The NO production inhibitory assay was performed as we previously reported [5]. The concentrations of TNF-α and IL-1β in RAW264.7 cell culture supernatants were quantified using Elisa kits according to the manufacturer’s instructions.

### 5.8. Supplementary Data

The Supplementary data including the NMR, HRESIMS spectra of compounds **1**–**4**, and cell viability assay was provided in Supplementary Materials.

## Figures and Tables

**Table 1:** ^1^H (600 MHz) and ^13^C (150 MHz) NMR data for compound **1** in CD_3_OD.

No.	δ_C_	δ_H_ (*J* in Hz)	No.	δ_C_	δ_H_ (*J* in Hz)
1	121.8	—	5′	77.8	3.56 (m)
2	114.0	7.62 (d, 1.8)	6′	67.8	3.66 (dd, 3.0, 12.0)
3	148.8	—			3.97 (dd, 1.8, 12.0)
4	153.4	—	1″	102.3	4.71 (d, 1.2)
5	116.0	6.86 (d, 8.4)	2″	72.1	3.83 (dd, 1.2, 9.6)
6	125.8	7.65 (dd, 1.8, 8.4)	3″	72.4	
7	166.7	—	4″	74.0	3.33 (dd, 9.6, 9.6)
1′	96.1	5.65 (d, 8.4)	5″	69.9	3.65 (m)
2′	74.1	3.48 (dd, 8.4, 9.6)	6″	18.0	1.19 (d, 6.6)
3′	78.1	3.49 (dd, 9.6, 9.6)	3-OCH_3_	56.5	3.91 (s)
4′	71.2				

There is no corresponding hydrogen data for quaternary carbons or carbonyl groups, they are represented as “—”.

**Table 2:** ^1^H (500 MHz) and ^13^C (125 MHz) NMR data for **2** in CD_3_OD.

No.	δ_C_	δ_H_ (*J* in Hz)	No.	δ_C_	δ_H_ (*J* in Hz)
1	130.2	—	4′	71.8	3.27 (dd, 9.0, 9.0)
2	113.3	7.03 (br. s)	5′	77.0	3.37 (m)
3	149.0	—	6′	68.2	3.64 (dd, 6.0, 11.0)
4	147.4	—			4.00 (br. d, ca. 11)
5	115.8	6.75 (d, 8.0)	1″	102.4	4.79 (br. s)
6	122.6	6.82 (br. d, ca. 8)	2″	72.3	3.87 (br. d, ca. 3)
7	71.2	4.54 (d, 11.5)	3″	72.4	3.69 (m, overlapped)
		4.77 (d, 11.5)	4″	74.1	3.38 (dd, 9.0, 9.5)
1′	102.7	4.29 (d, 8.0)	5″	69.9	3.69 (m, overlapped)
2′	75.1	3.22 (dd, 8.0, 8.5)	6″	18.1	1.27 (d, 6.5)
3′	78.1	3.31 (dd, 8.5, 9.0)	3-OCH_3_	56.4	3.86 (s)

**Table 3:** ^1^H and (600 MHz) ^13^C (150 MHz) NMR data for **3** in CD_3_OD.

No.	δ_C_	δ_H_ (*J* in Hz)	No.	δ_C_	δ_H_ (*J* in Hz)
1	66.1	4.21 (dd, 7.8, 11.4)	5′	76.9	3.35 (m)
		4.25 (dd, 7.8, 11.4)	6′	68.1	3.59 (dd, 6.0, 10.8)
2	121.5	5.36 (m)			3.98 (dd, 1.8, 10.8)
3	139.0	—	1″	102.3	4.75 (d, 1.2)
4	26.0	1.76 (s)	2″	72.2	3.83 (dd, 1.2, 3.6)
5	18.2	1.70 (s)	3″	72.4	3.65 (dd, 3.6, 9.6)
1′	102.5	4.26 (d, 7.8)	4″	74.1	3.36 (dd, 9.6, 9.6)
2′	75.1	3.16 (dd, 7.8, 9.0)	5″	69.8	3.66 (m)
3′	78.2	3.33 (dd, 9.0, 9.0)	6″	18.1	1.26 (d, 6.0)
4′	71.8	3.26 (dd, 9.0, 9.6)			

**Table 4:** ^1^H (500 MHz) and ^13^C (125 MHz) NMR data for **4** in CD_3_OD.

No.	δ_C_	δ_H_ (*J* in Hz)	No.	δ_C_	δ_H_ (*J* in Hz)
1	113.9	5.11 (br. d, ca. 11)	10	78.9	3.25 (m, overlapped)
		5.26 (br. d, ca. 17)	11	73.8	—
2	142.8	6.05 (dd, 11.0, 17.0)	12	24.9	1.12 (s)
3	77.2	—	13	25.9	1.16 (s)
4	89.9	3.53 (dd, 3.5, 9.0)	14	24.5	1.27 (s)
5	31.2	2.16 (ddd, 7.5, 7.5, 15.0)	15	16.5	1.60 (s)
		2.35 (ddd, 3.5, 7.5, 15.0)	1′	106.2	4.45 (d, 8.0)
6	123.7	5.53 (dd, 7.5, 7.5)	2′	75.9	3.23 (dd, 8.0, 9.0)
7	136.3	3.33 (dd, 9.0, 9.0)	3′	78.3	3.35 (dd, 9.0, 9.0)
8	37.9	2.05 (ddd, 8.5, 8.5, 14.0)	4′	71.6	3.33 (dd, 9.0, 9.5)
		2.24 (ddd, 4.0, 8.5, 14.0)	5′	78.0	3.25 (m, overlapped)
9	30.5	1.36 (m)	6′	62.8	3.71 (dd, 5.0, 12.0)
		1.75 (m)			3.86 (dd, 2.0, 12.0)

**Table 5:** Inhibitory effects of compounds **1**–**18** on NO production in RAW264.7 cells.

No.	NRC (%)	No.	NRC (%)	No.	NRC (%)
Normal	2.2 ± 0.2	**5**	99.8 ± 4.9	**12**	95.0 ± 2.3
Control	100 ± 3.0	**6**	93.0 ± 3.9	**13**	97.3 ± 3.8
DEX	76.8 ± 3.8 ***	**7**	95.0 ± 4.3	**14**	98.5 ± 2.2
**1**	91.7 ± 3.5 *	**8**	95.6 ± 3.1	**15**	91.2 ± 4.9 *
**2**	94.1 ± 3.7	**9**	93.9 ± 4.7	**16**	94.5 ± 3.4
**3**	96.7 ± 4.2	**10**	88.1 ± 3.2 ***	**17**	98.1 ± 3.4
**4**	90.2 ± 3.5 **	**11**	93.6 ± 2.8	**18**	94.2 ± 2.9

Positive control: Dexamethasone (Dex). Nitrite relative concentration (NRC): percentage of control group (set as 100%). Values represent the mean ± SD of six determinations. * *p* < 0.05; ** *p* < 0.01; *** *p* < 0.001 (Differences between compound-treated group and control group). Final concentration was 50 μM for compounds **1**–**18** and 1.0 μg/mL for Dex, respectively.

## Data Availability

The author does not guarantee the provision of original data.

## Supplementary Materials

The following supporting information can be downloaded at: https://www.mdpi.com/article/10.3390/molecules29051136/s1, Supplementary data including the NMR, HRESIMS spectra of compounds **1**–**4**, and cell viability assay associated with this article can be found in the online version.
