# Supplementary material for: Potential Anti-Inflammatory Constituents from Aesculus wilsonii Seeds
Source: Molecules. 2024 Mar 3;29(5):1136. doi: 10.3390/molecules29051136 (PMC10934013; doi:10.3390/molecules29051136)
Supplement: Supplementary file 1 [file molecules-29-01136-s001.zip › molecules-2840171-supplementary.pdf]

Supporting Information

Article

Bioactive Constituents from *Aesculus wilsonii* Seeds

Ping Zhang [1], Lequan Yu [1], Huina Cao [1], Jingya Ruan [2], Fei Li [1], Lijie Wu [1], Yi Zhang [1,2,*] and Tao Wang [1,2,*]

[1] Tianjin Key Laboratory of TCM Chemistry and Analysis, Tianjin University of Traditional Chinese Medicine, 10 Poyanghu Road, West Area, Tuanbo New Town, Jinghai District, Tianjin 301617, China; zp10259611@163.com (P.Z.); 18822575726@163.com (L.Y.); sxcaonanayy@163.com (H.C.); lf18641423285@163.com (F.L.); wulj0816@163.com (L.W.)

[2] Institute of TCM, Tianjin University of Traditional Chinese Medicine, 10 Poyanghu Road, West Area, Tuanbo New Town, Jinghai District, Tianjin 301617, China; ruanjingya@ tjutcm.edu.cn

* Correspondence: zhwwxzh@tjutcm.edu.cn (Y.Z.); wangtao@tjutcm.edu.cn (T.W.); Tel./Fax: +86-22-5959-6168 (T.W.)

Figure S1. ^1H NMR (600 MHz, CD_3OD) spectrum of **1** 3
Figure S2. ^{13}C NMR (150 MHz, CD_3OD) spectrum of **1** 3
Figure S3. ^1H ^1H COSY (CD_3OD) spectrum of **1** 4
Figure S4. HSQC (CD_3OD) spectrum of **1** 4
Figure S5. HMBC (CD_3OD) spectrum of **1** 5
Figure S6. ESI-Q-Orbitrap-MS spectrum of **1** 5
Figure S7. ^1H NMR (600 MHz, CD_3OD) spectrum of **2** 6
Figure S8. ^{13}C NMR (150 MHz, CD_3OD) spectrum of **2** 6
Figure S9. ^1H ^1H COSY (CD_3OD) spectrum of **2** 7
Figure S10. HSQC (CD_3OD) spectrum of **2** 7
Figure S11. HMBC (CD_3OD) spectrum of **2** 8
Figure S12. ESI-Q-Orbitrap-MS spectrum of **2** 8
Figure S13. ^1H NMR (600 MHz, CD_3OD) spectrum of **3** 9
Figure S14. ^{13}C NMR (125 MHz, CD_3OD) spectrum of **3** 9
Figure S15. ^1H ^1H COSY (CD_3OD) spectrum of **3** 10
Figure S16. HSQC (CD_3OD) spectrum of **3** 10
Figure S17. HMBC (CD_3OD) spectrum of **3** 11
Figure S18. ESI-Q-Orbitrap-MS spectrum of **3** 11
Figure S19. ^1H NMR (600 MHz, CD_3OD) spectrum of **4** 12
Figure S20. ^{13}C NMR (150 MHz, CD_3OD) spectrum of **4** 12
Figure S21. ^1H ^1H COSY (CD_3OD) spectrum of **4** 13
Figure S22. HSQC (CD_3OD) spectrum of **4** 13
Figure S23. HMBC (CD_3OD) spectrum of **4** 14
Figure S24. NOESY (CD_3OD) spectrum of **4** 14
Figure S25. ESI-Q-Orbitrap-MS spectrum of **4** 15
Figure S26. MTT assay of compounds **1–18** at the concentration of 50 μM on RAW264.7 cells 16

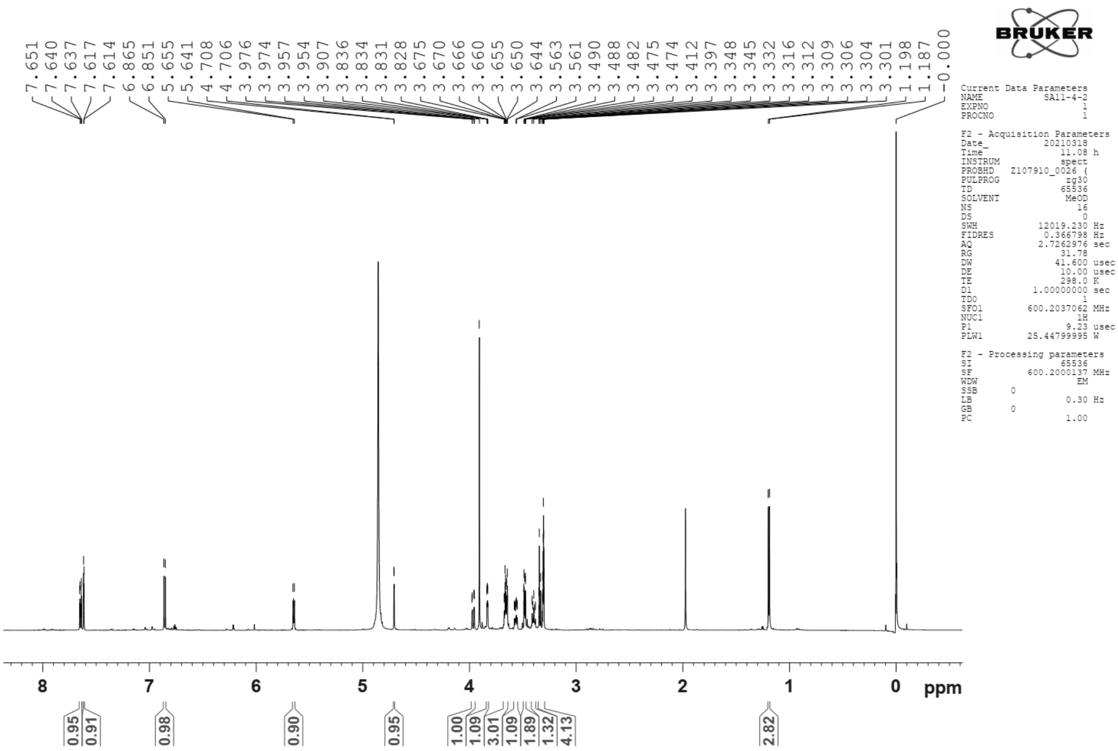

Figure S1. ^{1}H NMR (500 MHz, CD3OD) spectrum of **1**

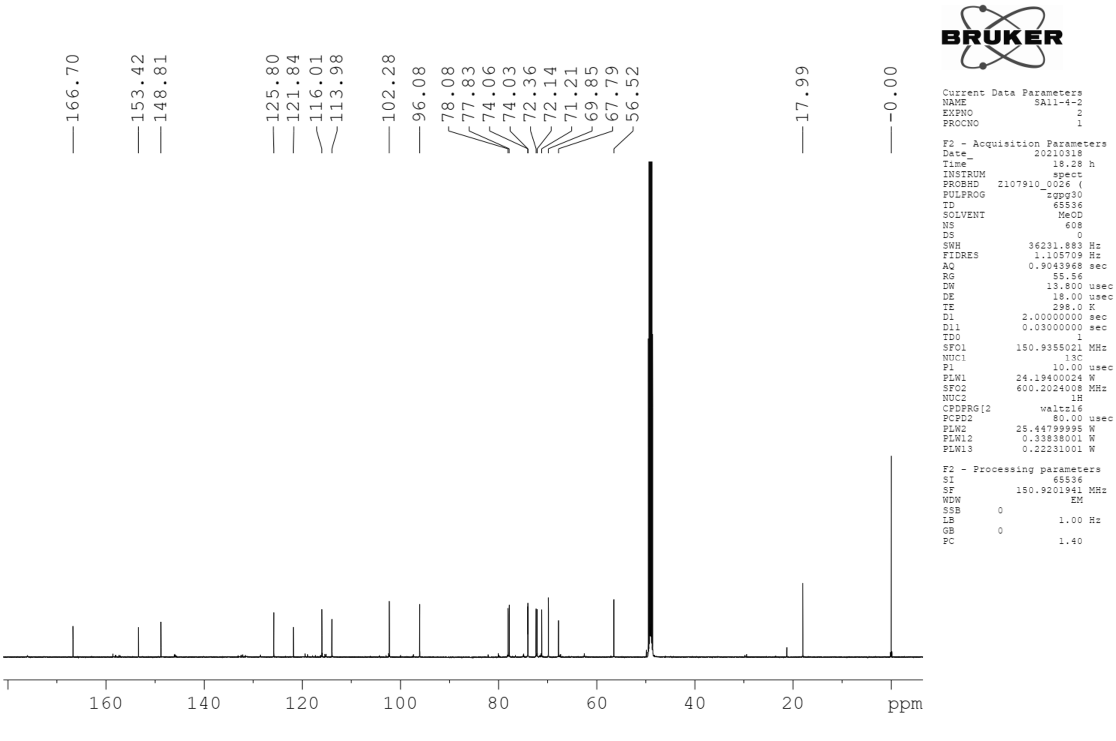

Figure S2. ^{13}C NMR (125 MHz, CD3OD) spectrum of **1**

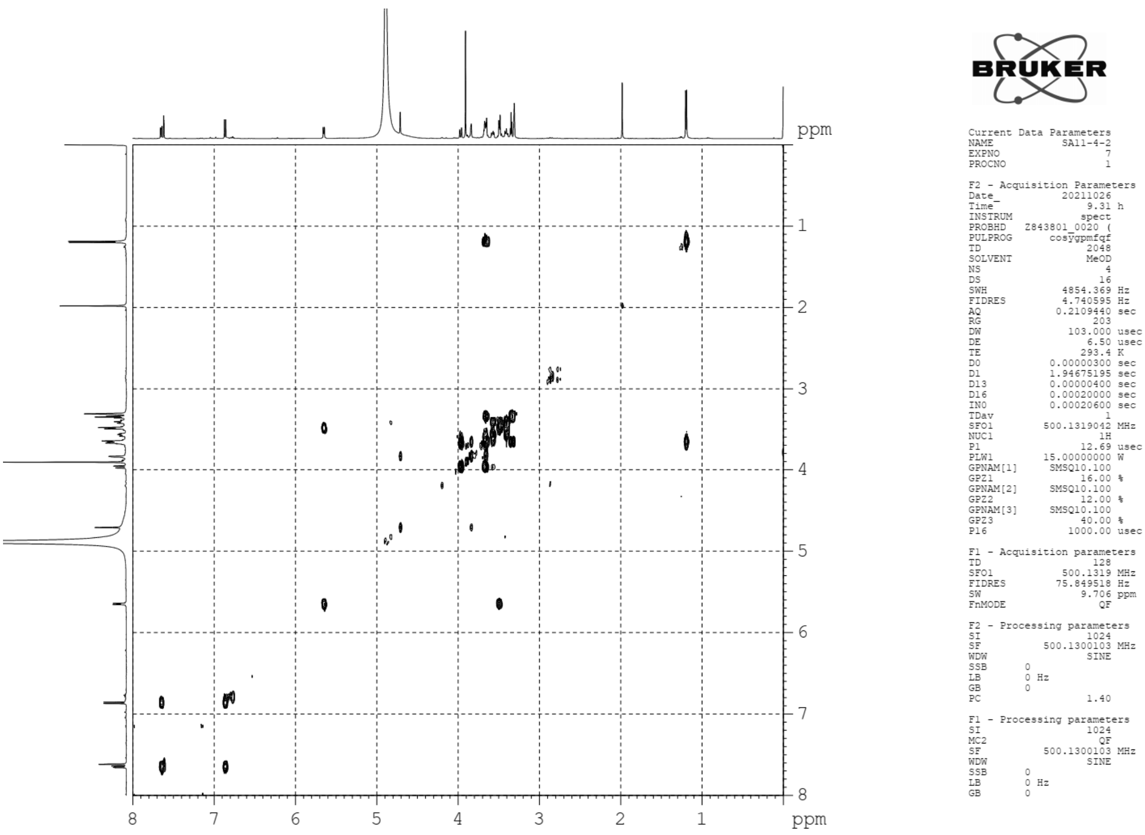

Figure S3. ^{1}H ^{1}H COSY (CD_3OD) spectrum of **1**

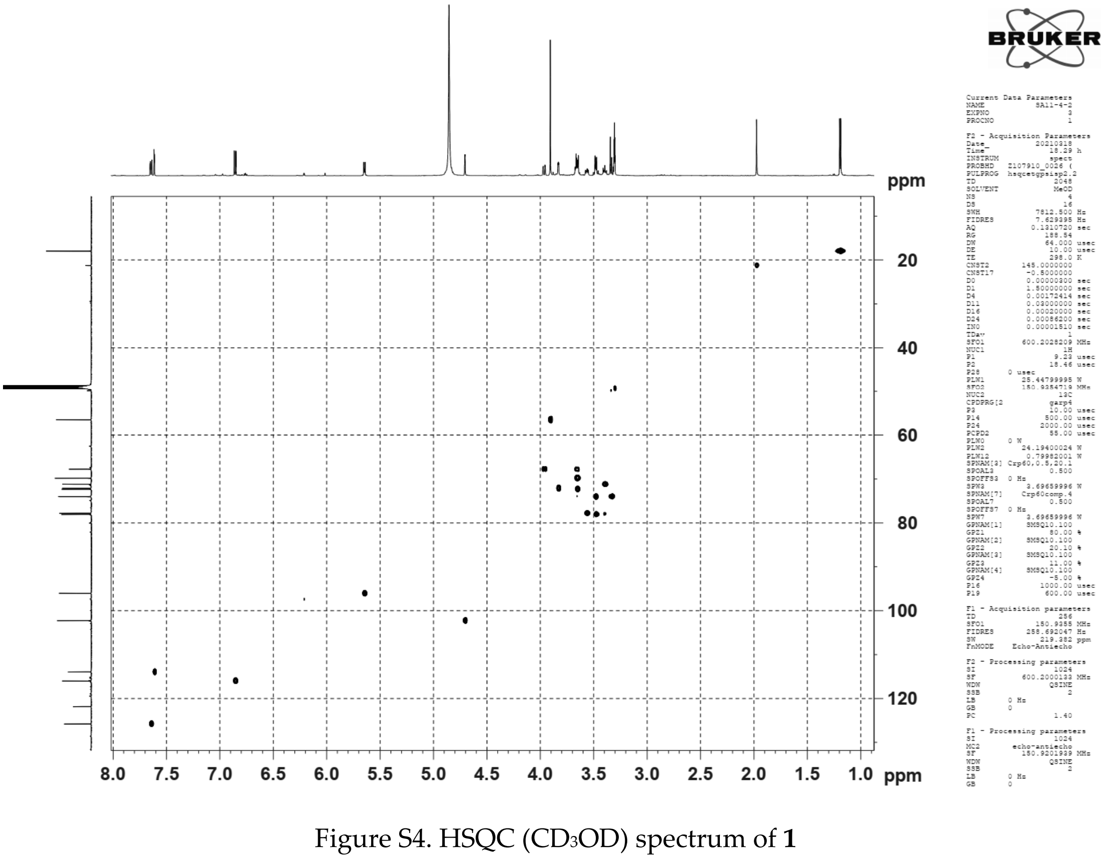

Figure S4. HSQC (CD_3OD) spectrum of **1**

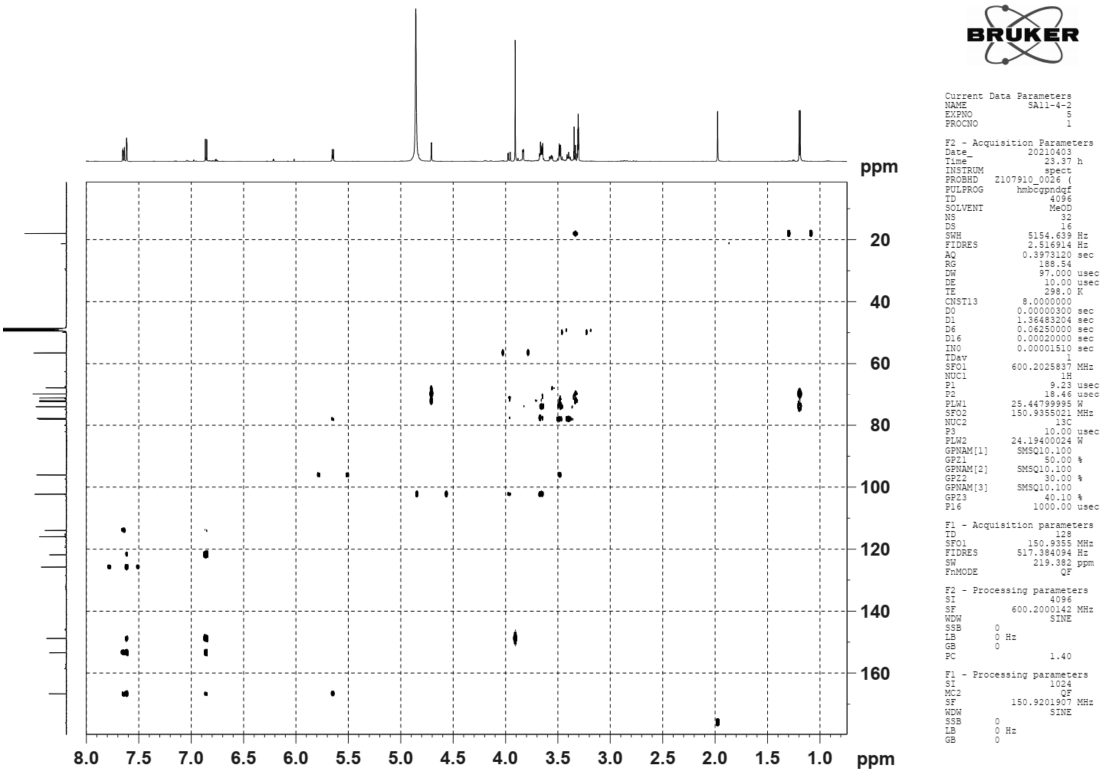

Figure S5. HMBC (CD_3OD) spectrum of **1**

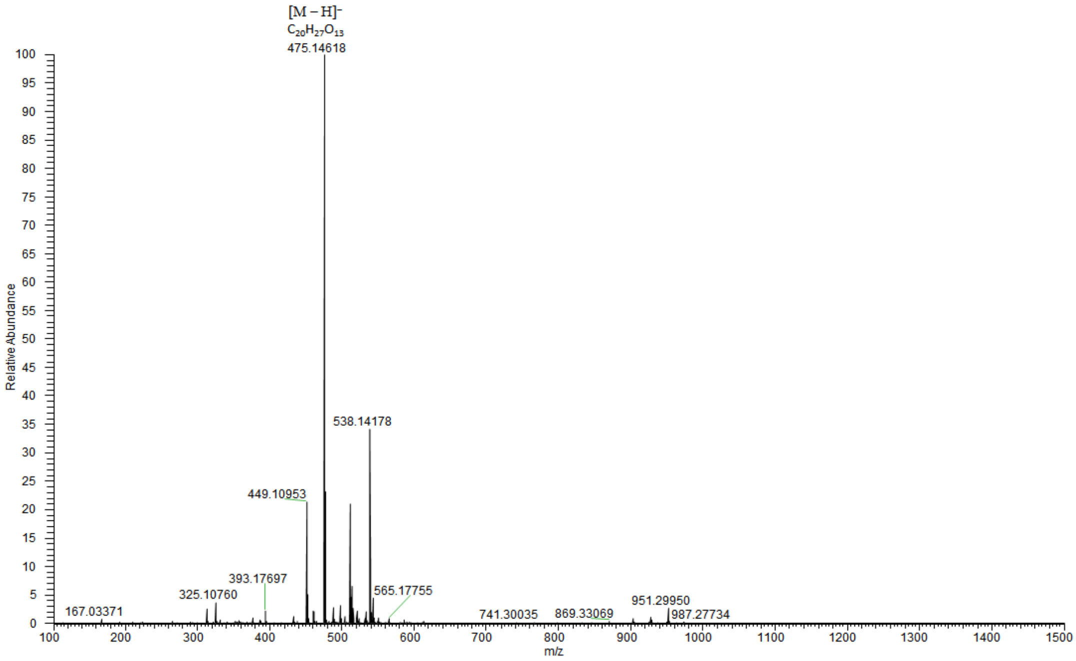

Figure S6. ESI-Q-Orbitrap-MS spectrum of **1**

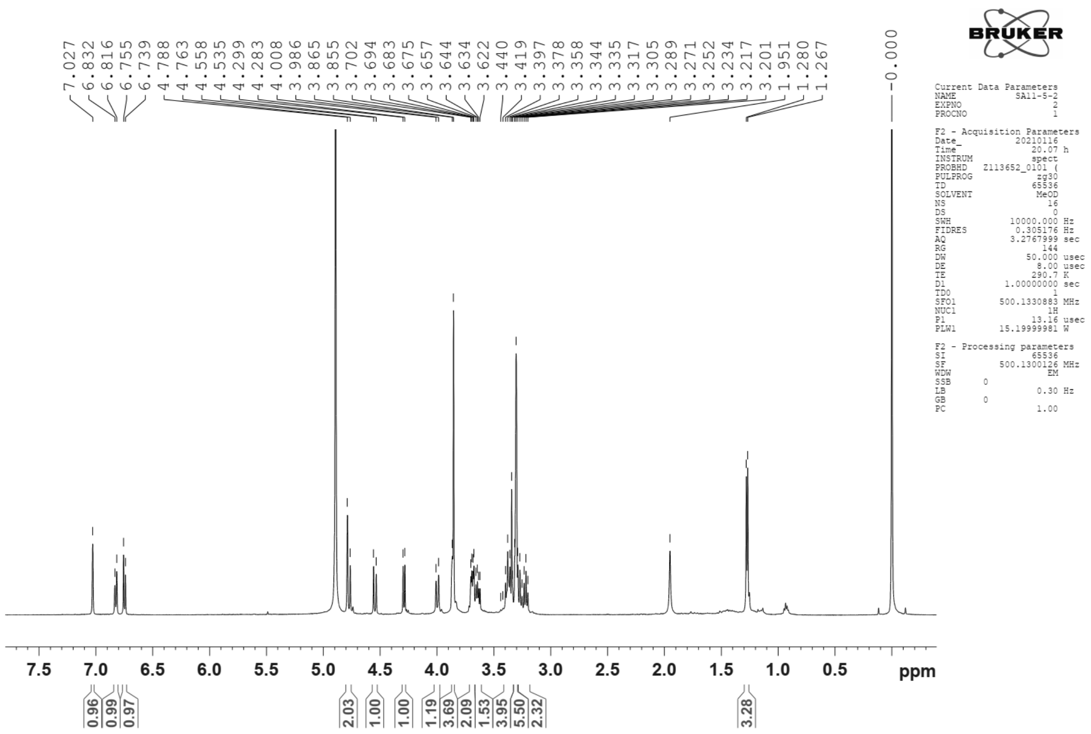

Figure S7. ^{1}H NMR (500 MHz, CD_3OD) spectrum of **2**

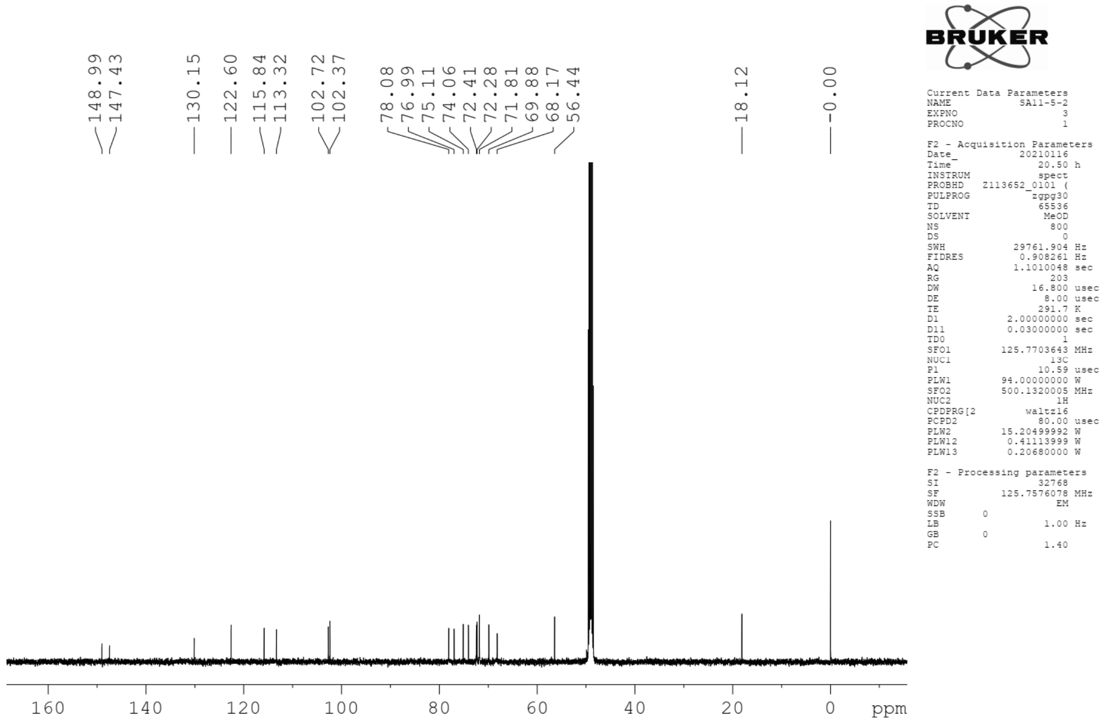

Figure S8. ^{13}C NMR (125 MHz, CD_3OD) spectrum of **2**

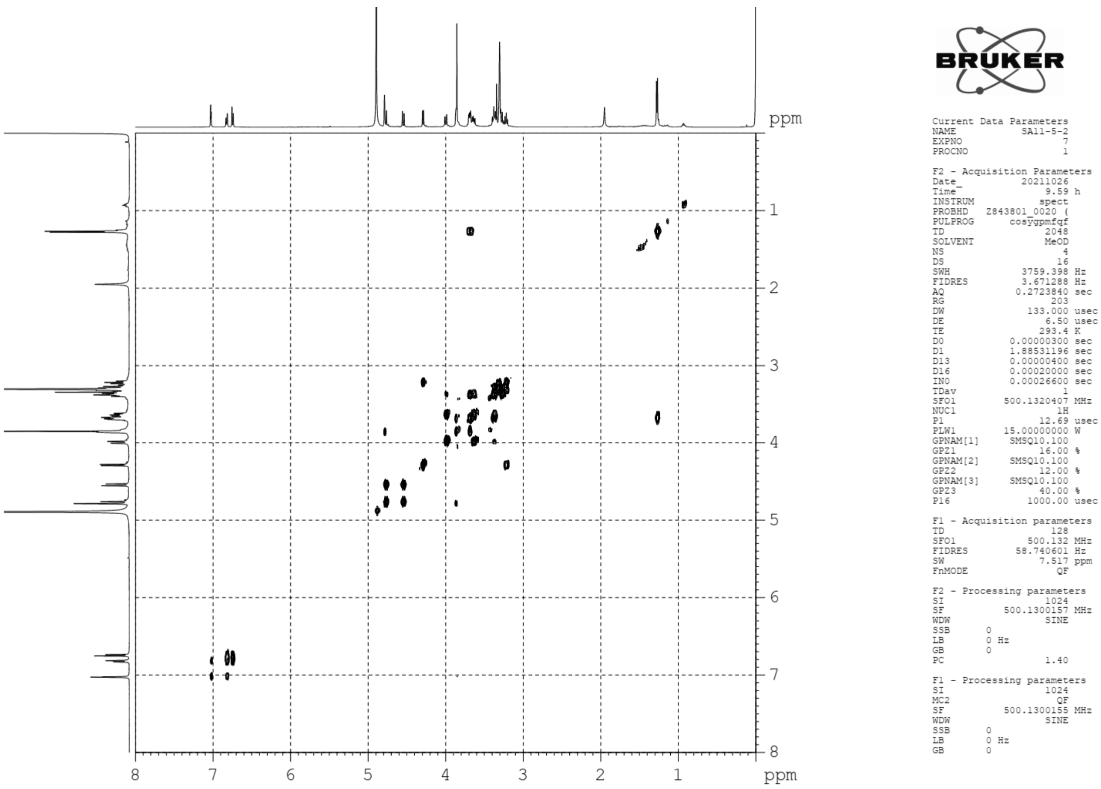

Figure S9. ^{1}H ^{1}H COSY (CD_3OD) spectrum of **2**

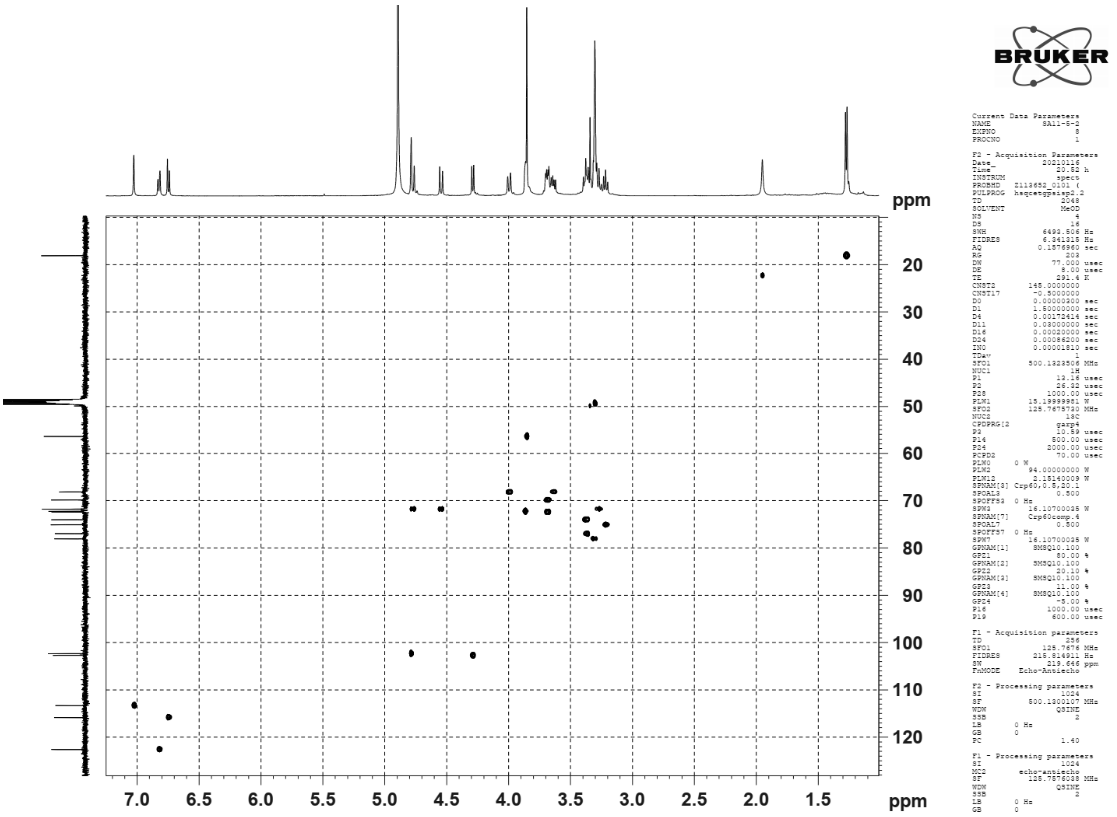

Figure S10. HSQC (CD_3OD) spectrum of **2**

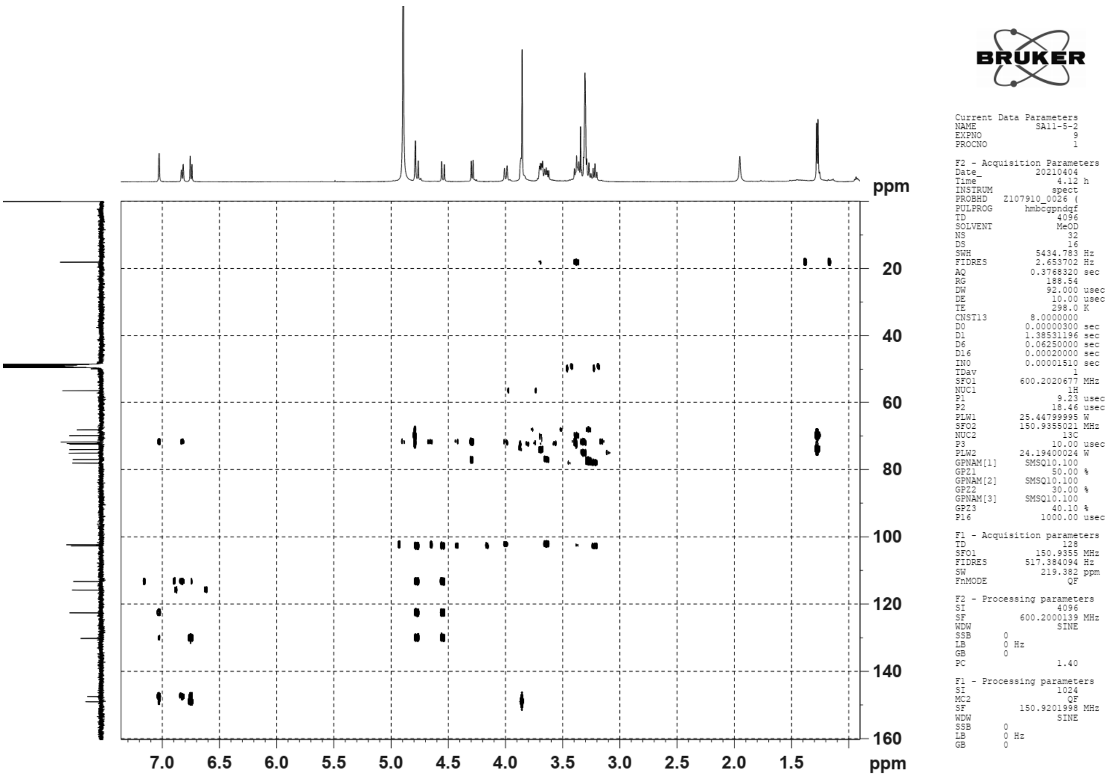

Figure S11. HMBC (CD_3OD) spectrum of **2**

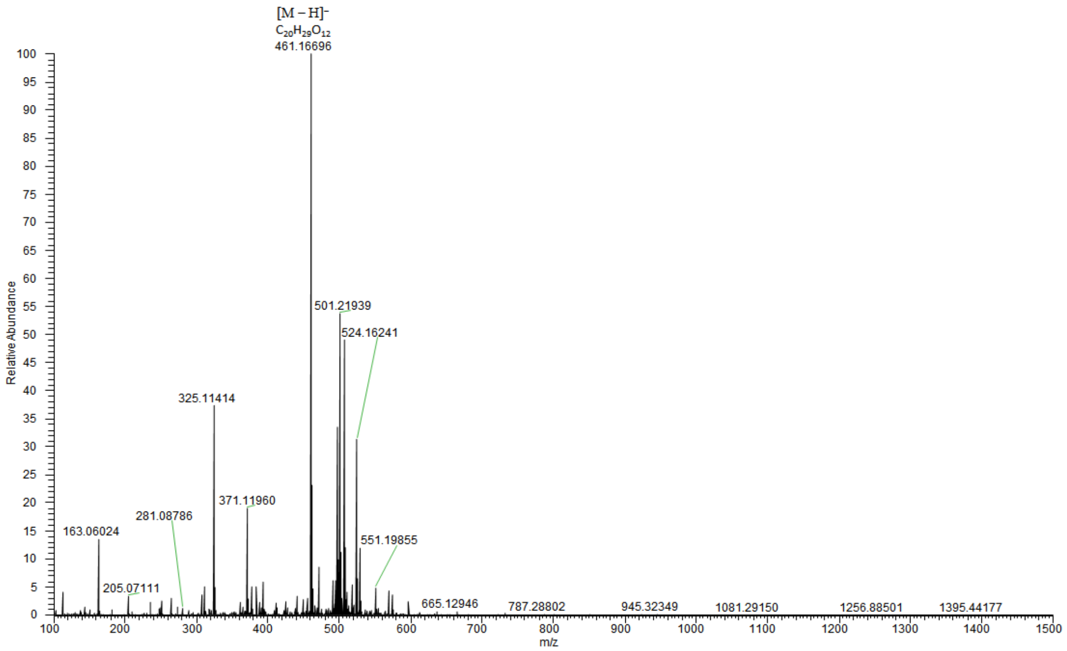

Figure S12. ESI-Q-Orbitrap-MS spectrum of **2**

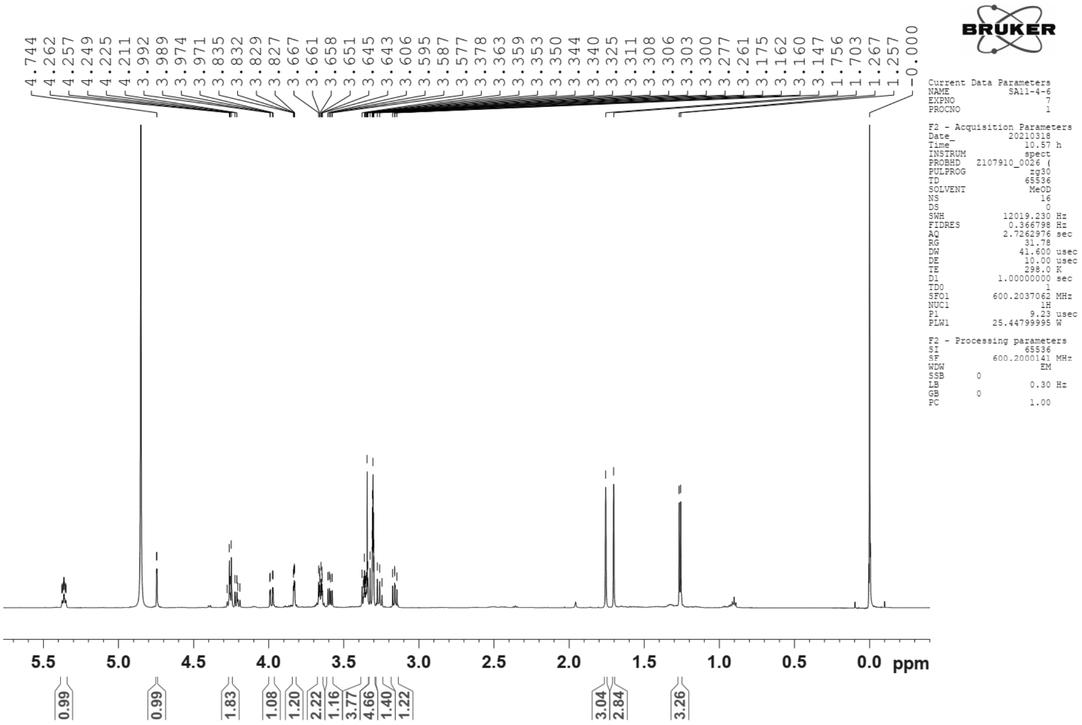

Figure S13. ¹H NMR (600 MHz, CD_3OD) spectrum of **3**

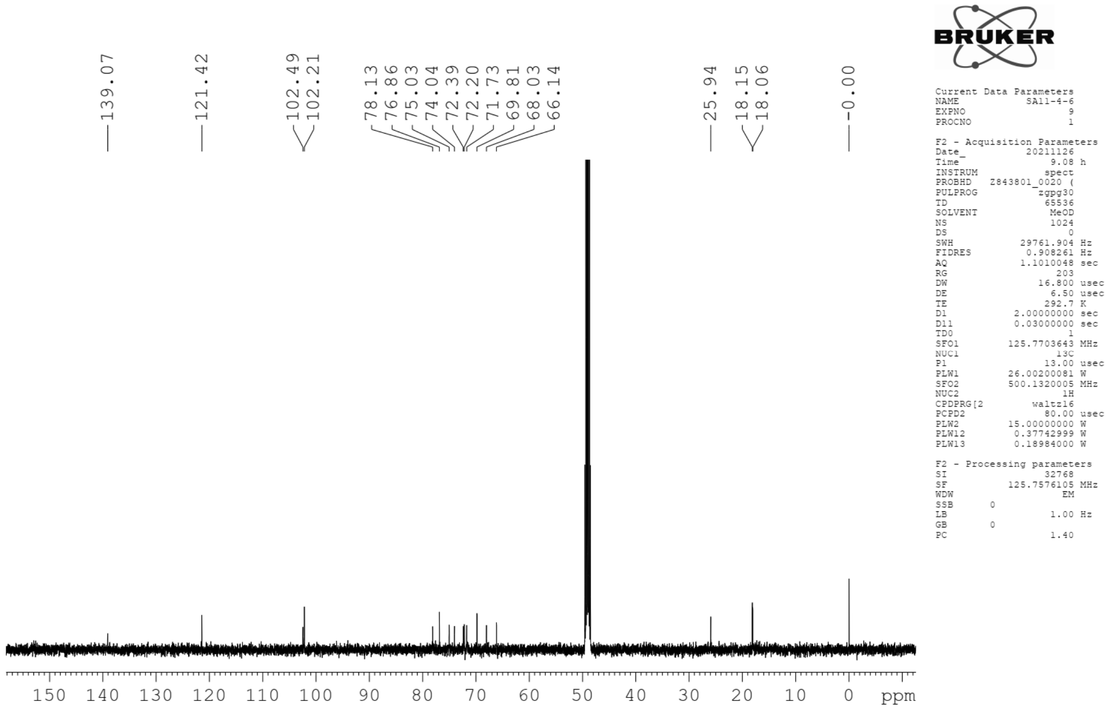

Figure S14. ¹³C NMR (125 MHz, CD_3OD) spectrum of **3**

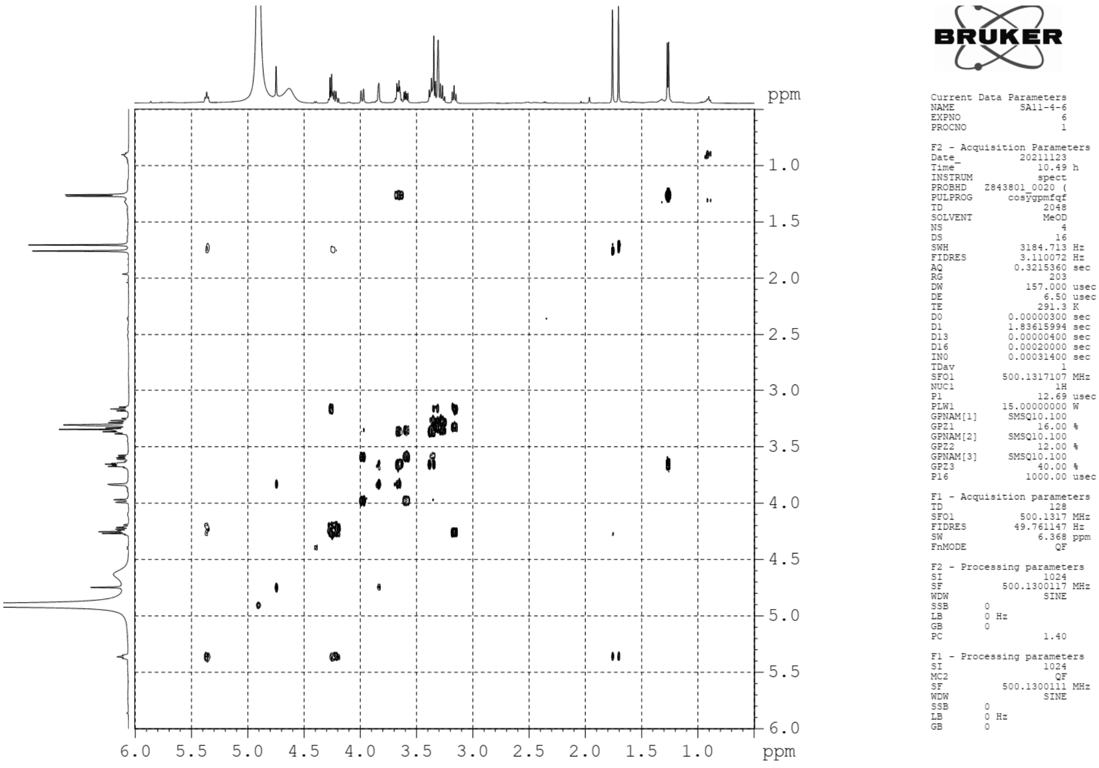

Figure S15. ^{1}H ^{1}H COSY (CD_3OD) spectrum of **3**

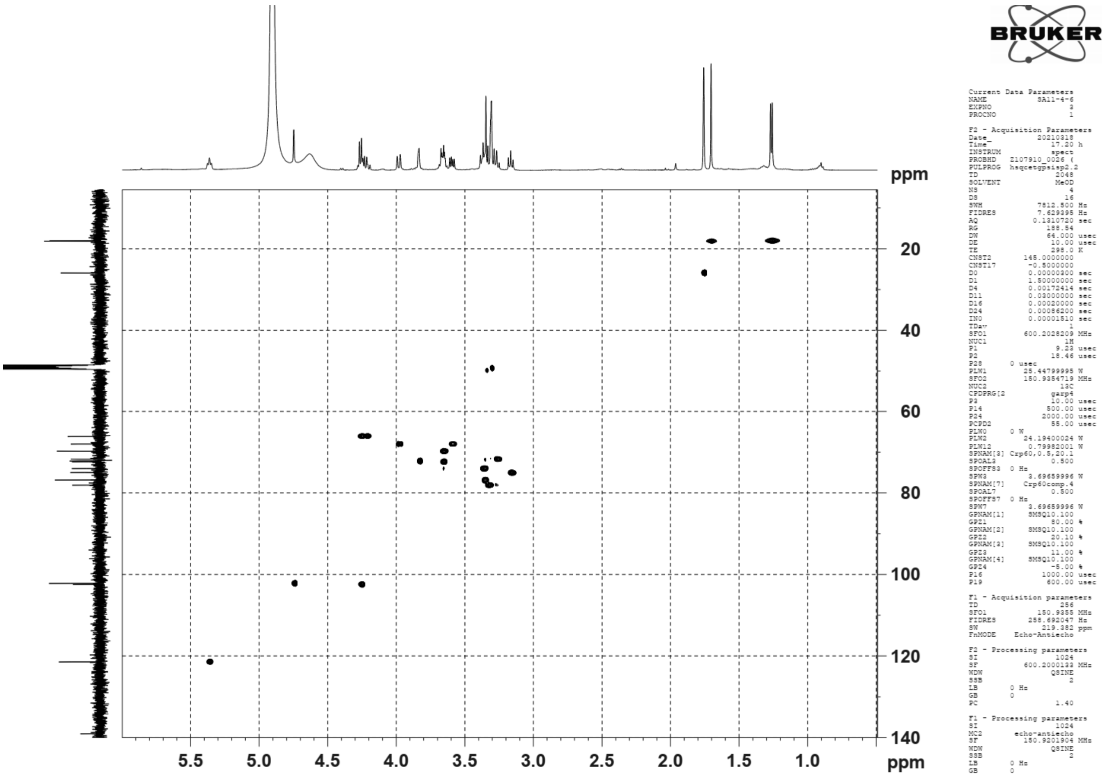

Figure S16. HSQC (CD_3OD) spectrum of **3**

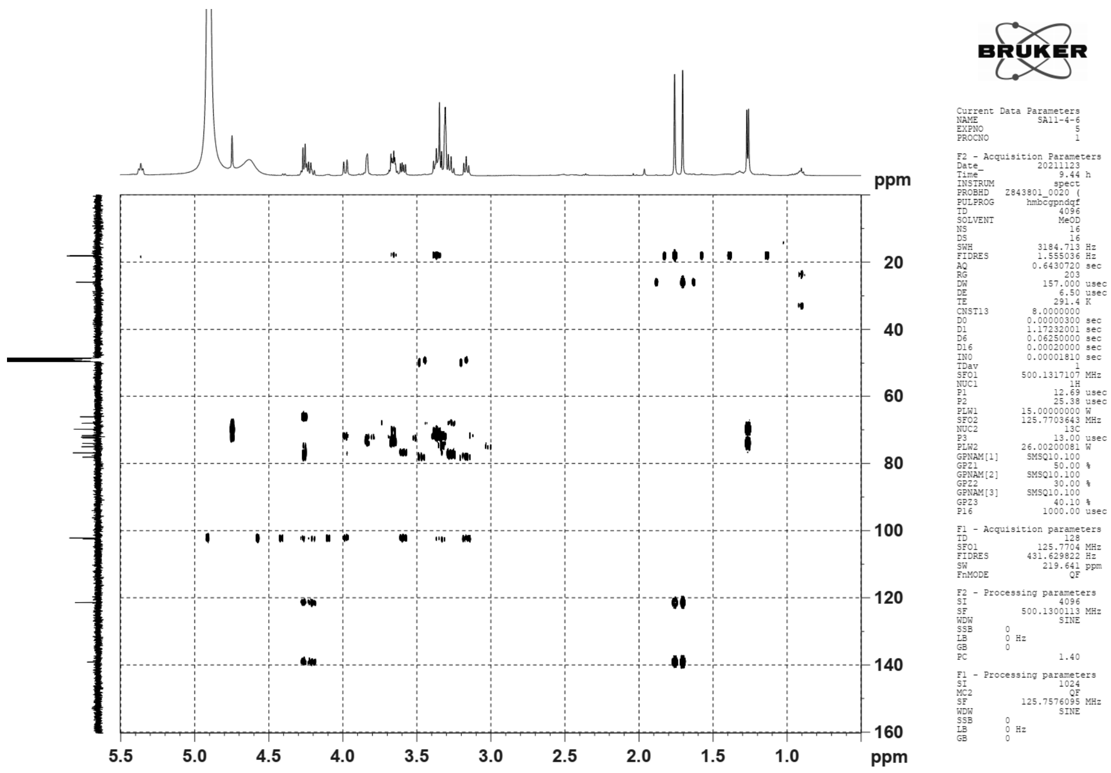

Figure S17. HMBC (CD_3OD) spectrum of **3**

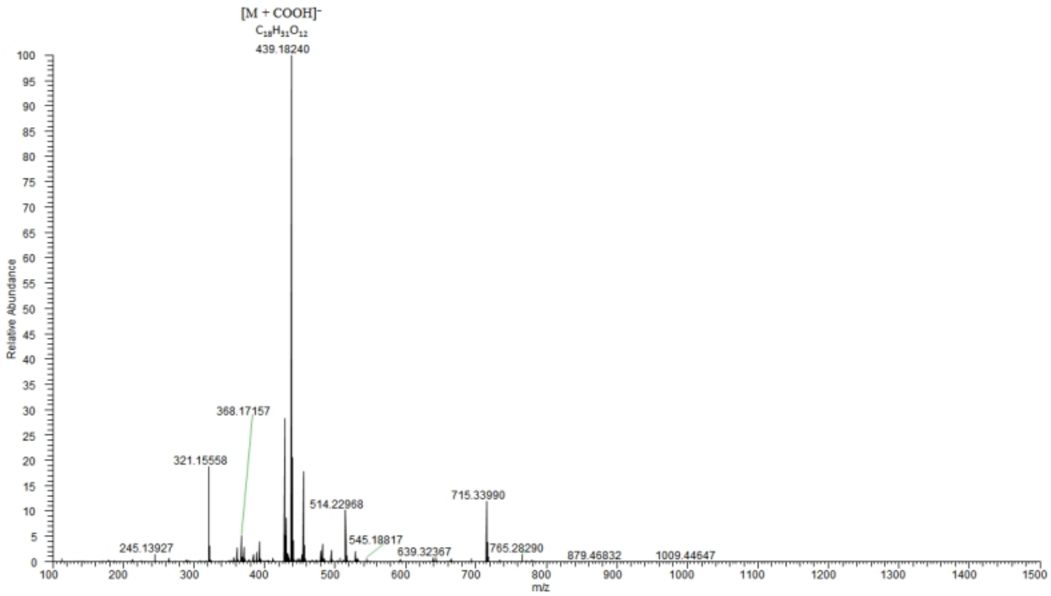

Figure S18. ESI-Q-Orbitrap-MS spectrum of **3**

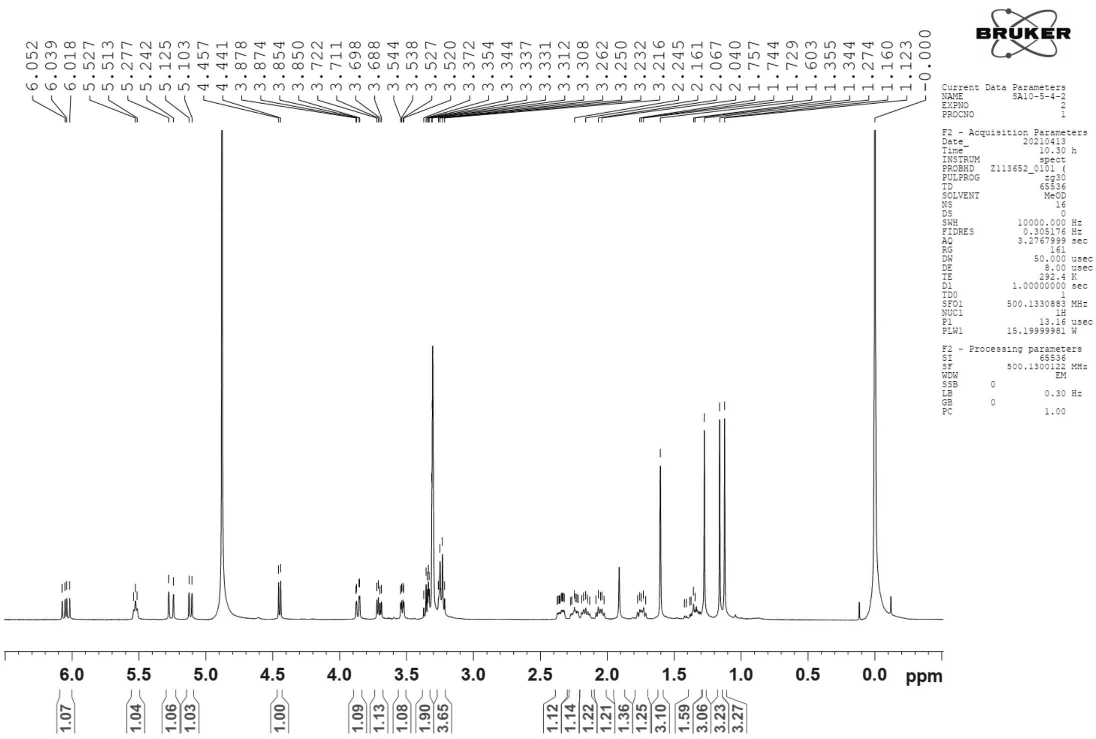

Figure S19. ^{1}H NMR (500 MHz, CD_3OD) spectrum of **4**

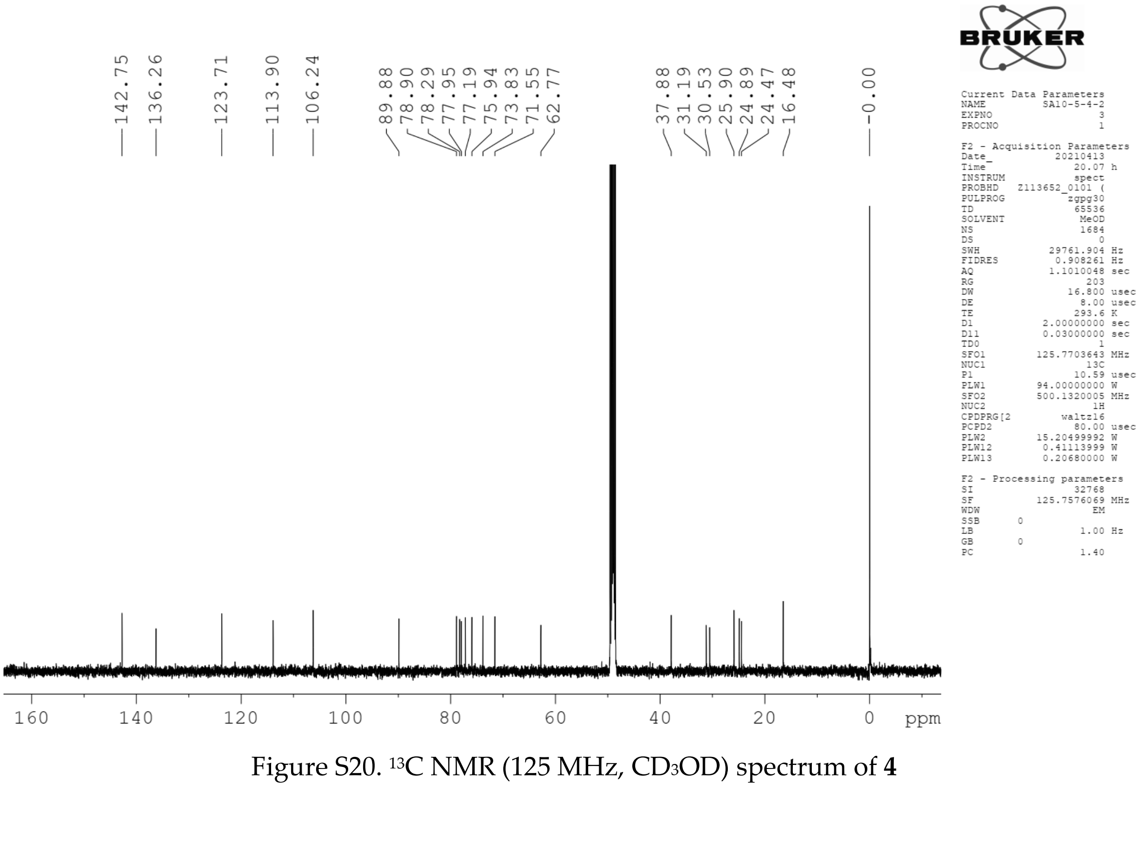

Figure S20. ^{13}C NMR (125 MHz, CD_3OD) spectrum of **4**

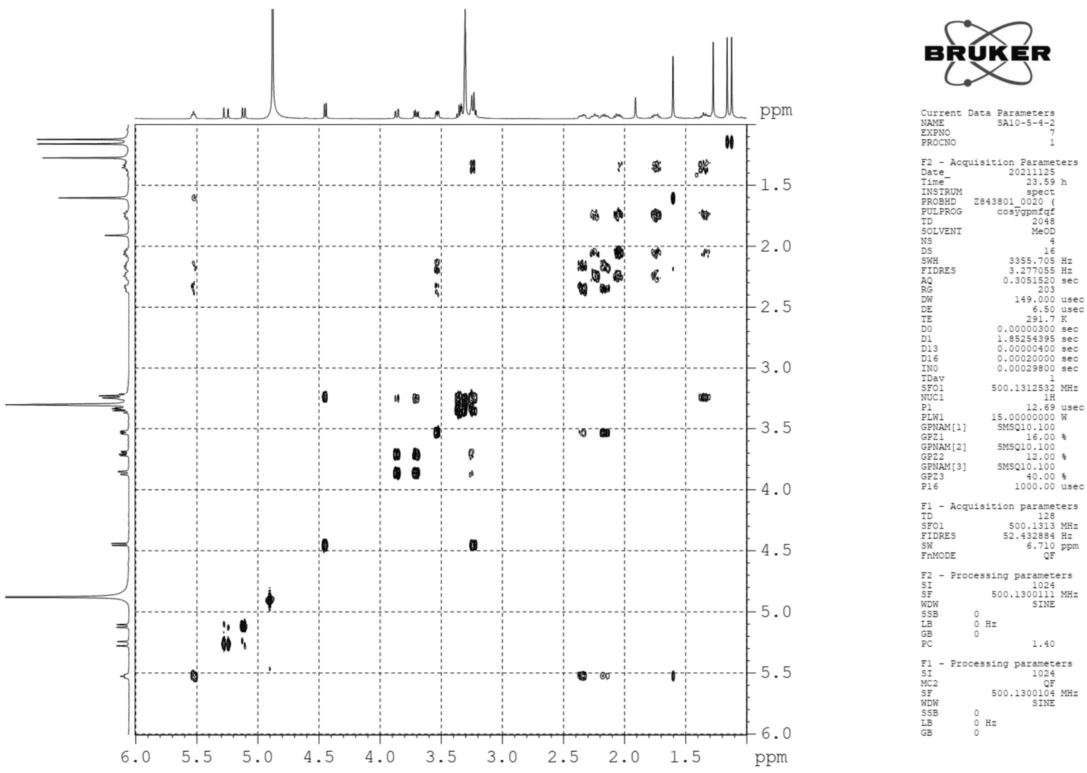

Figure S21. ^{1}H ^{1}H COSY (CD_3OD) spectrum of **4**

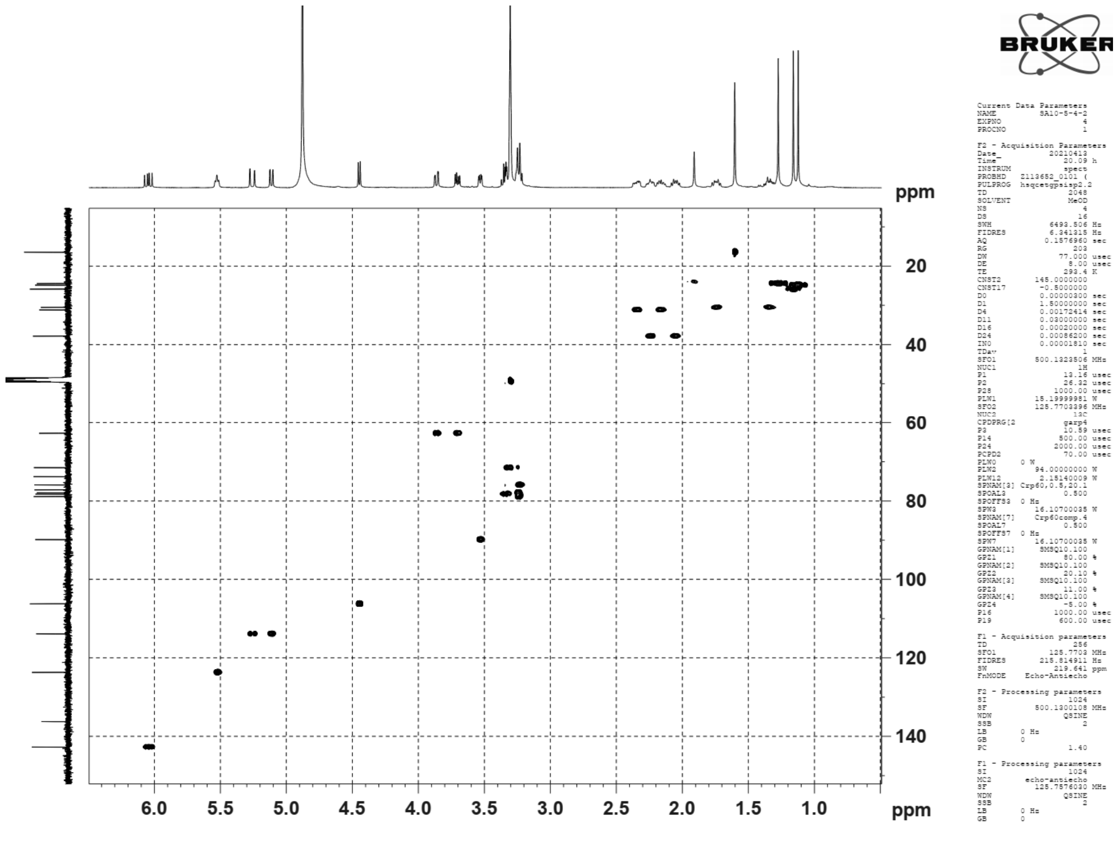

Figure S22. HSQC (CD_3OD) spectrum of **4**

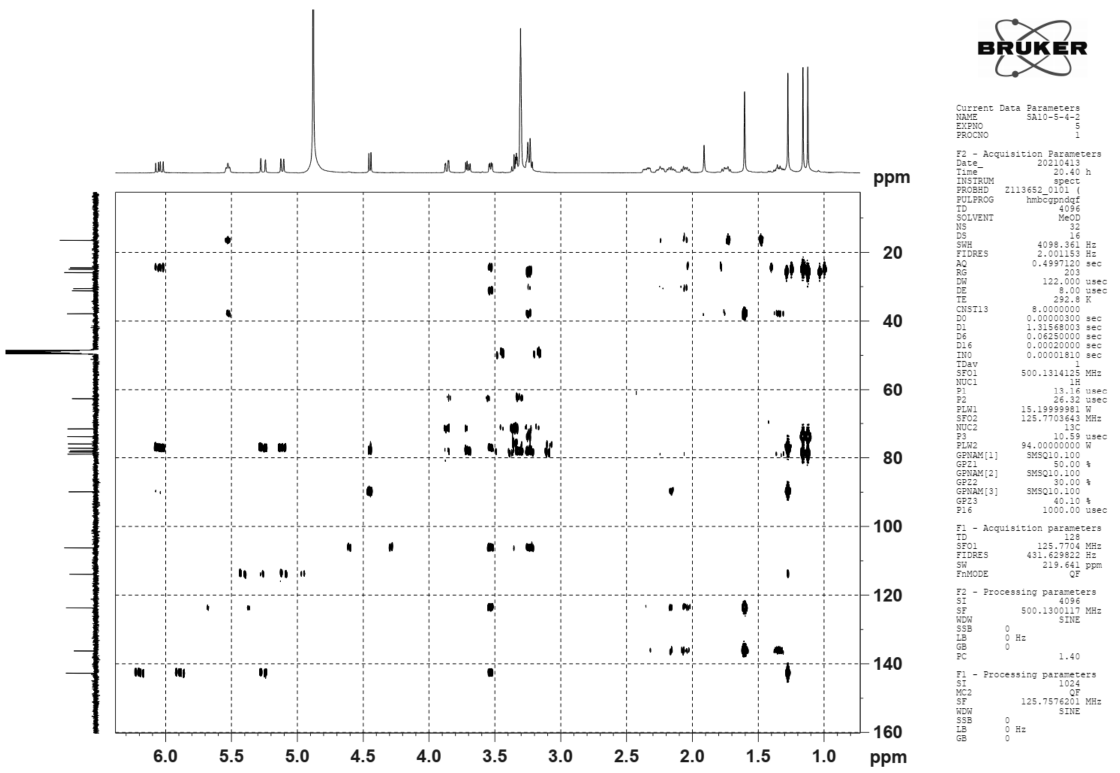

Figure S23. HMBC (CD3OD) spectrum of **4**

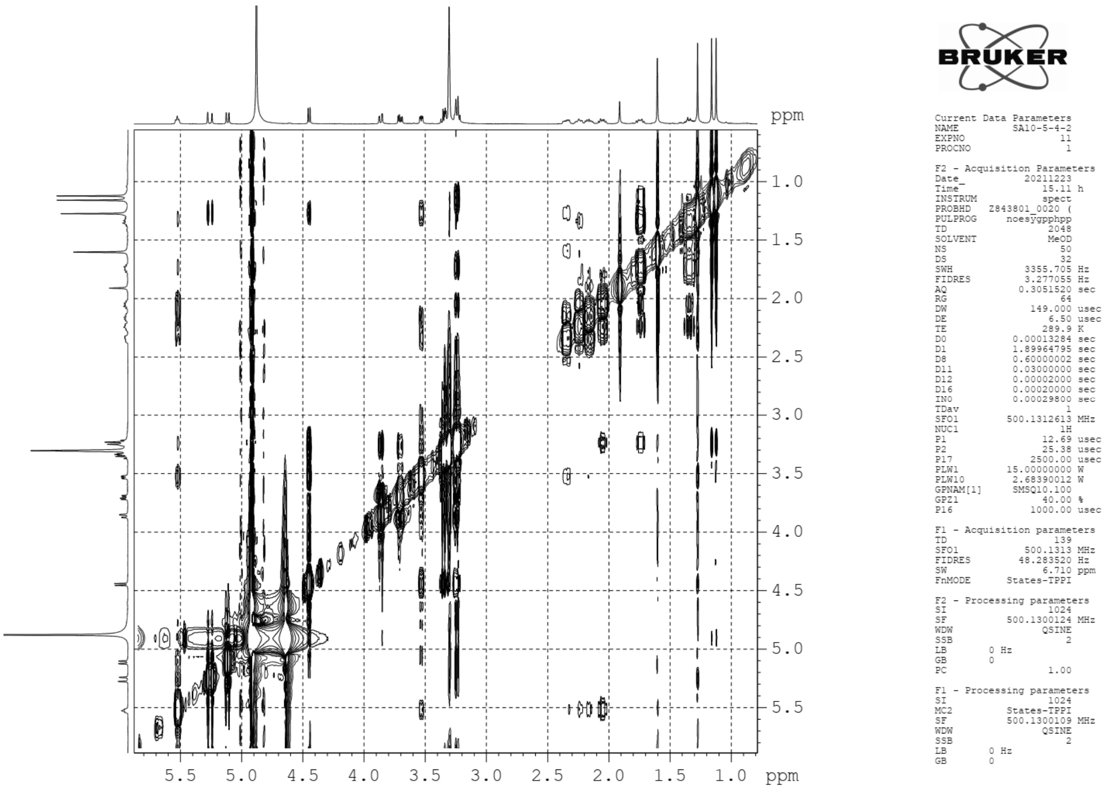

Figure S24. NOESY (CD3OD) spectrum of **4**

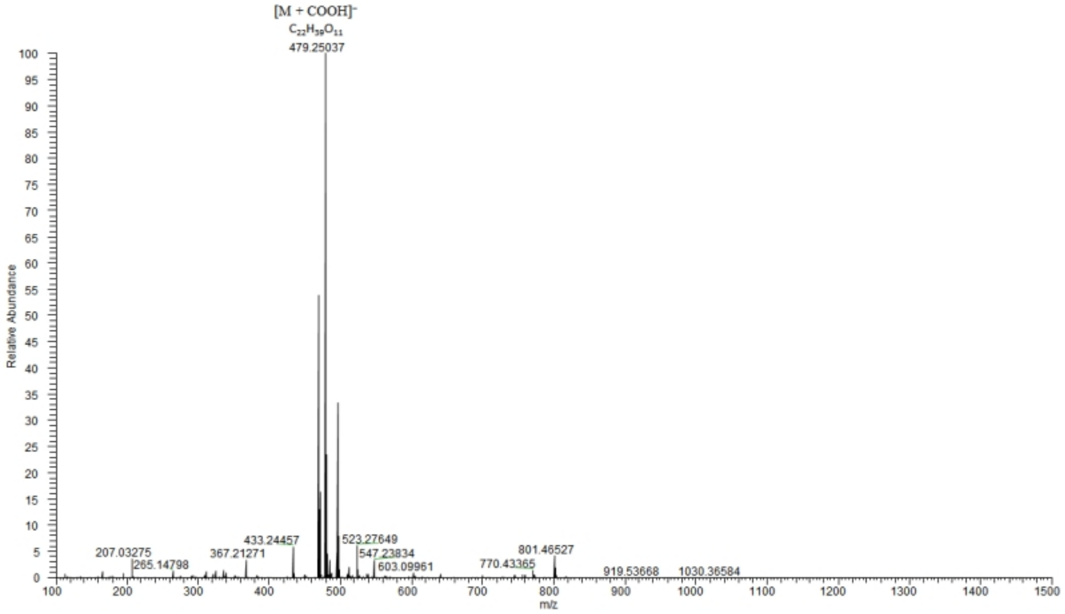

Figure S25. ESI-Q-Orbitrap-MS spectrum of **4**

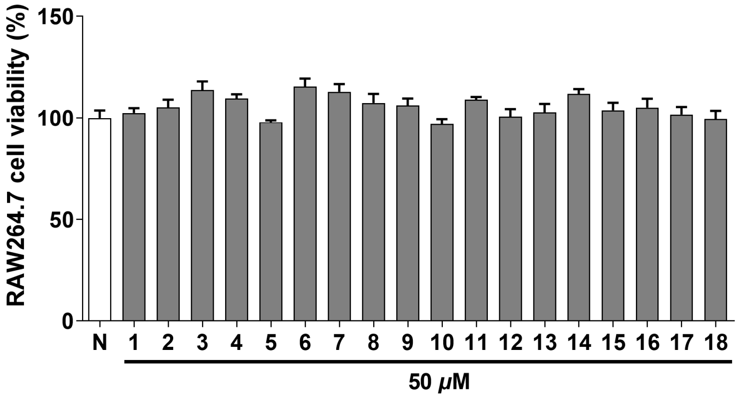

Figure S26. MTT asSAy of compounds **1–18** at the concentration of 50 μM on RAW264.7 cells
Cell viability: percentage of normal group (set as 100%). Values represent the mean ± SD of six determinations. ***P < 0.001 (Difference between compound-treated group and normal group).
